# Conserved Hydrocarbon Degradation Architectures in Petroleum-Associated Microbiomes Revealed by Genome-Resolved Analysis Using the Petroleum-Associated Genome Database (PaGeD)

**DOI:** 10.3390/microorganisms14102197

**Published:** 2026-10-01

**Authors:** Kelly Hidalgo-Martinez, Renato Augusto Corrêa dos Santos, João Vitor Leite Novoletti, Tulio de Lucca Capelini, Ana Letícia Malheiros Oliveira, Diego Mauricio Riaño-Pachón, German Zafra, Maira Paula de Sousa, Luiz Fernando Martins, Valéria Maia de Oliveira

**Affiliations:** 1 Divisão de Recursos Microbianos, Centro Pluridisciplinar de Pesquisas Químicas, Biológicas e Agrícolas (CPQBA), Universidade Estadual de Campinas—UNICAMP, Av. Alexandre Cazellato, 999, Paulínia 13148-218, SP, Brazil; 2 Laboratory of Computational, Evolutionary, and Systems Biology, Center for Nuclear Energy in Agriculture, University of São Paulo, Piracicaba 13416-000, SP, Brazil; renatoacsantos@gmail.com (R.A.C.d.S.); diego.riano@cena.usp.br (D.M.R.-P.); 3 Research Centre for Greenhouse Gas Innovation—RCG2I, São Paulo 05508-030, SP, Brazil; 4 National Institute for Science and Technology of Bioethanol, São Paulo 05508-090, SP, Brazil; 5 Laboratory of Isotopic Ecology, Center for Nuclear Energy in Agriculture, University of São Paulo, Piracicaba 13416-000, SP, Brazil; joao.novoletti@gmail.com; 6 Programa de Pós-Graduação em Genética e Biologia Molecular, Instituto de Biologia, Universidade Estadual de Campinas (UNICAMP), Rua Monteiro Lobato 255, Cidade Universitária, Campinas 13083-862, SP, Brazil; 7 Grupo de Investigación en Bioquímica y Microbiología (GIBIM), Escuela de Microbiología, Universidad Industrial de Santander, Cra 27 Calle 9, Bucaramanga 680002, Colombia; geralzaf@uis.edu.co; 8 PETROBRAS/R&D Center (CENPES), Av. Horácio Macedo, 950, Cidade Universitária, Ilha do Fundão, Rio de Janeiro 21941-915, RJ, Brazil; mpsousa@petrobras.com.br (M.P.d.S.); martinslf@petrobras.com.br (L.F.M.)

**Keywords:** microbiomes, genomics, petroleum, hydrocarbon, bioremediation

## Abstract

Microbial communities play key roles across the oil supply chain, driving both detrimental processes such as corrosion and souring and beneficial applications including bioremediation. However, genomic and functional information on these microorganisms remains fragmented, limiting integrative analyses. Here, we perform a global genome-resolved analysis of petroleum-associated microbiomes based on a curated collection of 3334 prokaryotic genomes from reservoirs, produced waters, hydrocarbon-polluted sites and related environments worldwide, integrated within the Petroleum-Associated Genome Database (PaGeD). These genomes cluster into 2522 species-level units, more than half representing previously undescribed taxa, revealing extensive hidden microbial diversity in petroleum systems. Functional profiling using 144 KEGG ortholog groups linked to hydrocarbon degradation uncovers a modular organization in which central catabolic pathways are widely conserved across habitats and phylogenetic groups, whereas substrate-specific pathways are restricted to fewer taxa. Gene–genome network analysis further reveals a gradient of functional richness, from generalist genomes occupying central positions to specialized degraders at the periphery. Despite pronounced geographic and ecological heterogeneity, degradation profiles are largely conserved, indicating convergent functional adaptation to hydrocarbon exposure. This work provides a genome-resolved framework for understanding the ecological organization and biotechnological potential of petroleum-associated microbiomes.

## 1. Introduction

The oil and gas industry is a crucial sector in the global economy, mainly due to fossil fuels still serving as the primary energy source [1]. Microorganisms play critical roles across this supply chain, mediating processes that can be both detrimental, such as microbial influenced corrosion, souring, and biofouling, and beneficial, like bioremediation and wastewater treatment. Oil reservoirs, storage tanks, pipelines, and related petroleum-associated environments are characterized by selective conditions, including high pressure, elevated temperature, high salinity, and high concentration of heavy metals and organic solvents, which strongly shape microbial persistence, activity, and community structure [2,3]. Nevertheless, microorganisms inhabiting these environments have demonstrated remarkable adaptability [4,5], supported in part by the presence of water in many stages of production systems as well as in reservoirs [2].

In this context, several fundamental questions arise: (i) Which microorganisms can grow under these extreme conditions? (ii) What genomic traits and metabolic strategies enable their persistence and ecological success? (iii) Which microbial functions of industrial relevance can be inferred from their genomes, particularly those associated with hydrocarbon transformation? (iv) Can these microorganisms be systematically identified and prioritized for future biotechnological applications within the oil supply chain?

Advances in large-scale DNA sequencing and bioinformatics have revolutionized our understanding of oil-associated microbiomes [2], overcoming many of the limitations of culture-based methods. Shotgun metagenomics now enables the reconstruction of near-complete genomes (metagenome-assembled genomes—MAGs) from complex microbial communities inhabiting produced and injection waters, oil-polluted soils, storage tanks, pipelines, and oil reservoirs. These genomic resources make it possible to explore, at unprecedent resolution, the taxonomic diversity and functional potential underlying microbial processes in petroleum-related environments.

While pioneering initiatives, such as the Genome Repository of Oil Systems (GROS) [6], have enriched our knowledge, these efforts have primarily focused on specific events, like the Deepwater Horizon spill, leaving a gap in comprehensive genomic resources that cover the entire oil supply chain.

To expand our knowledge of the taxonomy and functional potential of oil-associated microbiomes, we developed the Petroleum-Associated Genome Database (https://paged.cpqba.unicamp.br/). Here, we present an interactive web platform that organizes and provides access to curate genome-associated data, including taxonomic, ecological, and functional information, from petroleum-related microorganisms. PaGeD offers an easy-to-navigate interface with search capabilities, filters, and robust ways to interpret the genomic content as a function of, e.g., isolation source and metabolic traits. In addition to providing deeper insights into microbial diversity in extreme oil-associated habitats, PaGeD also represents a resource that can be leveraged in future investigations to help cope with the negative roles of microorganisms and/or develop products and processes of strategic interest for the security and sustainability of the oil and gas industry.

## 2. Material and Methods

### 2.1. Genome Collection and Metadata Retrieval

To explore the diversity of prokaryotic genomes in the oil and gas industry, we searched for publicly available genomes from environments such as oil reservoirs, produced or formation water, fracturing fluids, deep seeps, and contaminated soils or waters. We retrieved genome data from major databases, including Integrated Microbial Genomes & Microbiomes (IMG/M) [7], National Center for Biotechnology Information (NCBI) [8], and the Genome Repository of Oil Systems (GROS) via the Microbial Genome Atlas [6,9]. Additional genomes not deposited in these repositories were identified through targeted searches in scientific literature databases (e.g., Google Scholar, Scopus). For this, we used combinations of keywords such as “oil”, “oil field”, “oil reservoir”, “petroleum”, “crude oil”, “injection water”, “formation water”, “produced water”, “production water”, “oil refinery” and “microbially influenced corrosion” to retrieve relevant studies. When genome files or metadata were not publicly available, we contacted the original authors directly to request access to the corresponding data. All searches and data retrieval were completed by February 2024. All accession numbers and associated publications for the genomes included in PaGeD are provided in Table S1.

Most metagenome-assembled genomes (MAGs) used in this study were retrieved as preassembled genome bins from these public repositories and were originally reconstructed by the respective authors from their own metagenomic datasets and pipelines. In addition, a subset of MAGs originates from studies conducted by our group [10,11,12] and were generated using a previously published and validated reconstruction pipeline [13]. No metagenomic assembly or binning was performed specifically for the present study; our contribution consisted of curating, quality-filtering, dereplicating, and functionally annotating these genomes and integrating them into a standardized framework.

**Figure 1:**
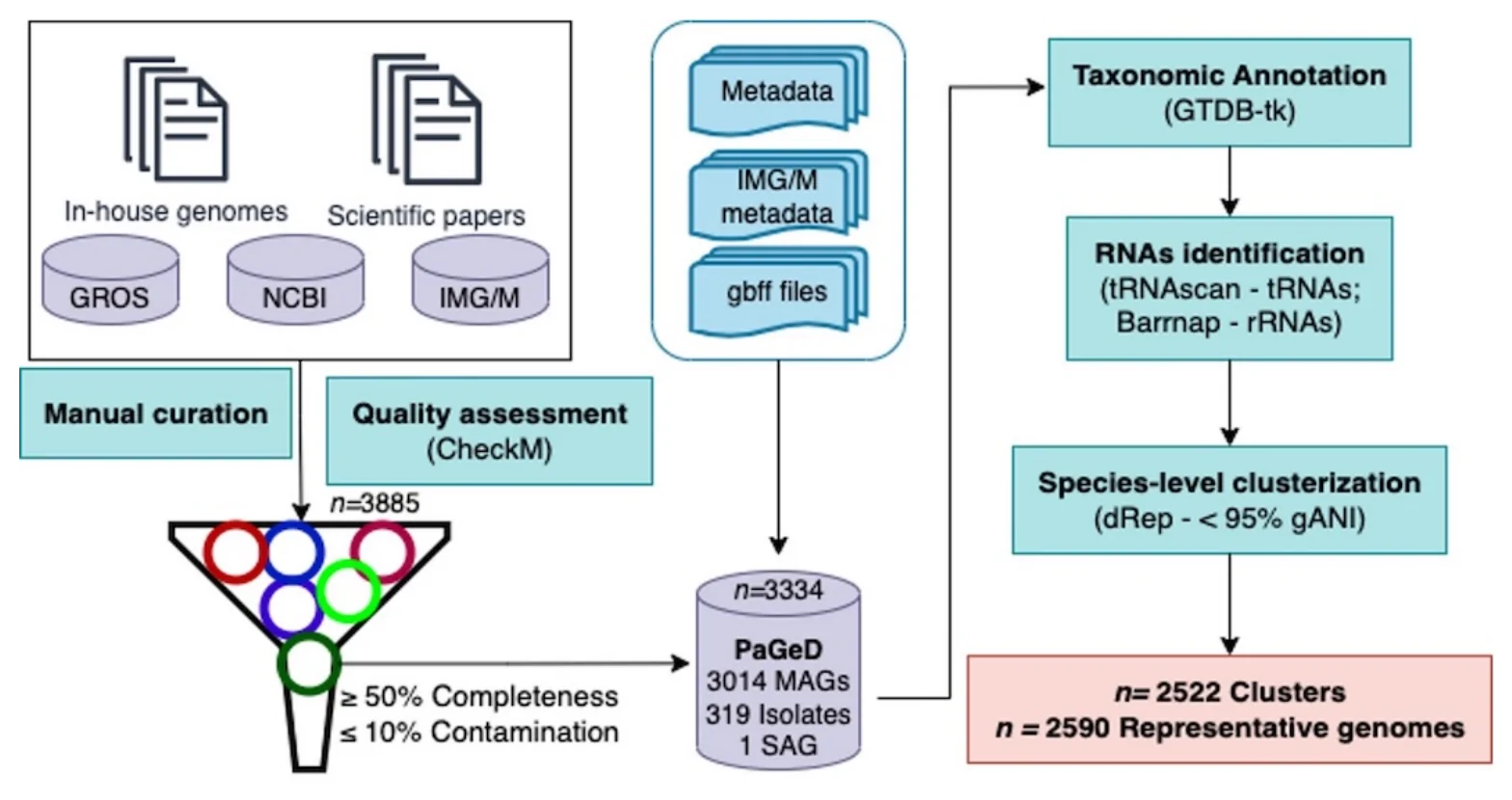
Workflow for genome retrieval, filtering, annotation, and dereplication to construct the PaGeD.

After manual curation to remove duplicate entries, 3885 genomes were compiled (Figure 1). Metadata for each genome, including geographic origin, geographic coordinates, and genome type (MAG or isolate), were extracted from the respective databases (Table S1). Habitat and isolation source categories were established based on metadata fields available in public repositories, particularly the “habitat” and isolation source” descriptors provided in the NCBI Biosample and JGI/IMG records. These terms were manually curated and harmonized into consistent categories which are fully documented in Table S2.

### 2.2. Quality Assessment and Taxonomic Classification

Quality assessment was performed using CheckM v1.1.3 (https://ecogenomics.github.io/CheckM/, Accessed on 10 March 2026, Parks et al., 2015), retaining only genomes with ≥50% completeness and <10% contamination (according to MiMAG standards; [14]). Furthermore, tRNAs were predicted with tRNAscan-SE v2.0.12 [15] and rRNAs identified using Barrnap v1.10.5 (https://github.com/tseemann/barrnap, Accessed on 10 March 2026). These steps yielded a final dataset of 3334 medium- and high-quality genomes. GTDB-tk v2.3.2 (Release 214.1, April 2023; [16]) was used for taxonomic annotation, and genomes were clustered into 2522 species-level groups (average nucleotide identity—ANI >95% and >30% alignment coverage [17]) with representative genomes selected using dRep v3.4.3 [18]. For phylogenetic reconstruction, genomes harboring at least 30% of the marker genes were filtered and then processed with GToTree v1.7 [19], and the resulting tree was visualized using iTOL v6 [20].

### 2.3. Functional Annotation

Open reading frames (ORFs) were predicted with Prodigal v2.6.3. The ORFs were then annotated using the sequence aligner Diamond v.0.9.30 [21] based on the KEGG database (Kyoto Encyclopedia of Genes and Genomes) [22]. Hits were retained based on the stringent confidence thresholds (sequence identity ≥ 60% and alignment coverage ≥ 60% of query length).

Hydrocarbon degradation-related functions were determined through profiling all the genomes against a curated set of 144 KEGG Orthology (KO) identifiers associated with hydrocarbon degradation pathways (Table S3). This curated list comprised genes involved in hydrocarbon activation, peripheral degradation reactions, and central aromatic-intermediate metabolism. Genes encoding enzymes involved in the initial activation of pathway-entry streps, such as oxygenases and other hydrocarbon activating enzymes, were considered strong indicators of hydrocarbon degradation potential. Genes involved in the downstream metabolism of aromatic intermediates, including catechol, benzoate, and benzoyl-CoA pathways, were interpreted as indicators of the capacity to process degradation intermediates and to integrate these compounds into the central aromatic metabolism.

For each genome, the pathway/module completeness score was calculated as the proportion of detected KOs relative to the total number of curated KOs assigned to each hydrocarbon degradation-related pathway or module. This score was used as a comparative measure of KO-set representation, rather than as a binary inference of a fully functional pathway. Alternative enzymes, optional branches, and multiple biochemical routes were not explicitly resolved as separate Boolean pathway structures; instead, all curated KOs assigned to a given pathway/module were retained in the denominator. Therefore, completeness values should be interpreted as a fraction of curated pathway-associated KOs detected in each genome or species-level cluster. Genome completeness, including MAG completeness, was not used to normalize pathway scores, and missing KOs were therefore interpreted cautiously, as they may reflect either true absence or incomplete genome recovery.

### 2.4. Bipartite Co-Occurrence Network

To explore the functional organization of hydrocarbon degradation genes across genomes, we constructed a bipartite network linking KEGG Orthologs (KOs) to the genomes encoding them. This network was not intended to infer unknown gene–genome associations, since genes were already assigned to their corresponding genomes through the annotation process. Instead, it was used as an exploratory framework to visualize observed KO–genome associations, summarize how hydrocarbon degrading-related functions are distributed across genomes, identify highly connected functional hubs, and examine gradients of functional specialization among genomes with hydrocarbon degradation-related potential.

Only genomes encoding ≥15 distinct hydrocarbon degradation KOs were retained for network construction. Bipartite edges were defined by the presence of a given KO in a genome, yielding a genome–gene network in which nodes represent either genomes or genes and edges represent gene occurrence. In parallel, a KO–KO co-occurrence matrix was computed from a binary KO-by-genome matrix and retained for downstream analyses, but visualization focused on the bipartite structure to preserve the direct mapping between functions and hosts.

Gene nodes were classified by their frequency across genomes: rare (<5%), intermediate (5–15%) or core (≥15%). Functional richness was defined as the number of distinct hydrocarbon degradation KOs encoded per genome and treated as a continuous measure positioning genomes along a gradient from specialist-like to generalist-like. For visualization and interpretation, genomes in the upper quartile of functional richness were referred to as generalist-like, whereas genomes in the lower quartile were referred to specialist-like; genomes with intermediate values occupied transitional positions along this gradient.

The network was built using the igraph v1.5.1 R package and visualized with ggraph v2.1.0. Gene nodes were displayed as hollow symbols, colored by pathway class and shaped by frequency category, whereas genome nodes were displayed as filled circles, colored by phylum and scaled according to functional richness. This representation highlights conserved catabolic cores, rare and peripheral pathways, and the distribution of functional potential across genomes.

Basic topological properties of the resulting network, including the number of nodes and edges, degree distribution, and modularity, were calculated using igraph v1.5.1 and used to support the interpretation organization and hub structure.

### 2.5. Platform Implementation and Interface

The Petroleum-Associated Genome Database (PaGeD) was developed as an open-source web platform for interactive exploration of taxonomic, functional, and ecological data associated with oil microbiomes.

The platform comprises a Python Flask v3.0.0. and MariaDB v14.14 as the database engine, providing a lightweight and efficient means of storing and retrieving genomic data. To support fast and flexible search functionality, the platform integrates the Whooshee extension v2.7.4, enabling full-text search across genome attributes, taxonomic classification, and functional annotations.

The front end was built using HTML, CSS, and JavaScript, using Bootstrap for responsive design. Interactive visualizations were implemented with Chart.js v4.0 and Leaflet.js v2.0.0, complemented by jQuery v3.7.1 and qTip2.js v3.0.3 for enhanced interactivity. The platform includes pages for database overview, taxonomy explorer, functional explorer, and species-specific pages. Species pages integrate taxonomic classification, geographic mapping, habitat information, and genome metadata, including accession numbers from public repositories (e.g., NCBI, JGI).

## 3. Results and Discussion

### 3.1. Global Environmental Distribution of the PaGeD Genomes

**Figure 2:**
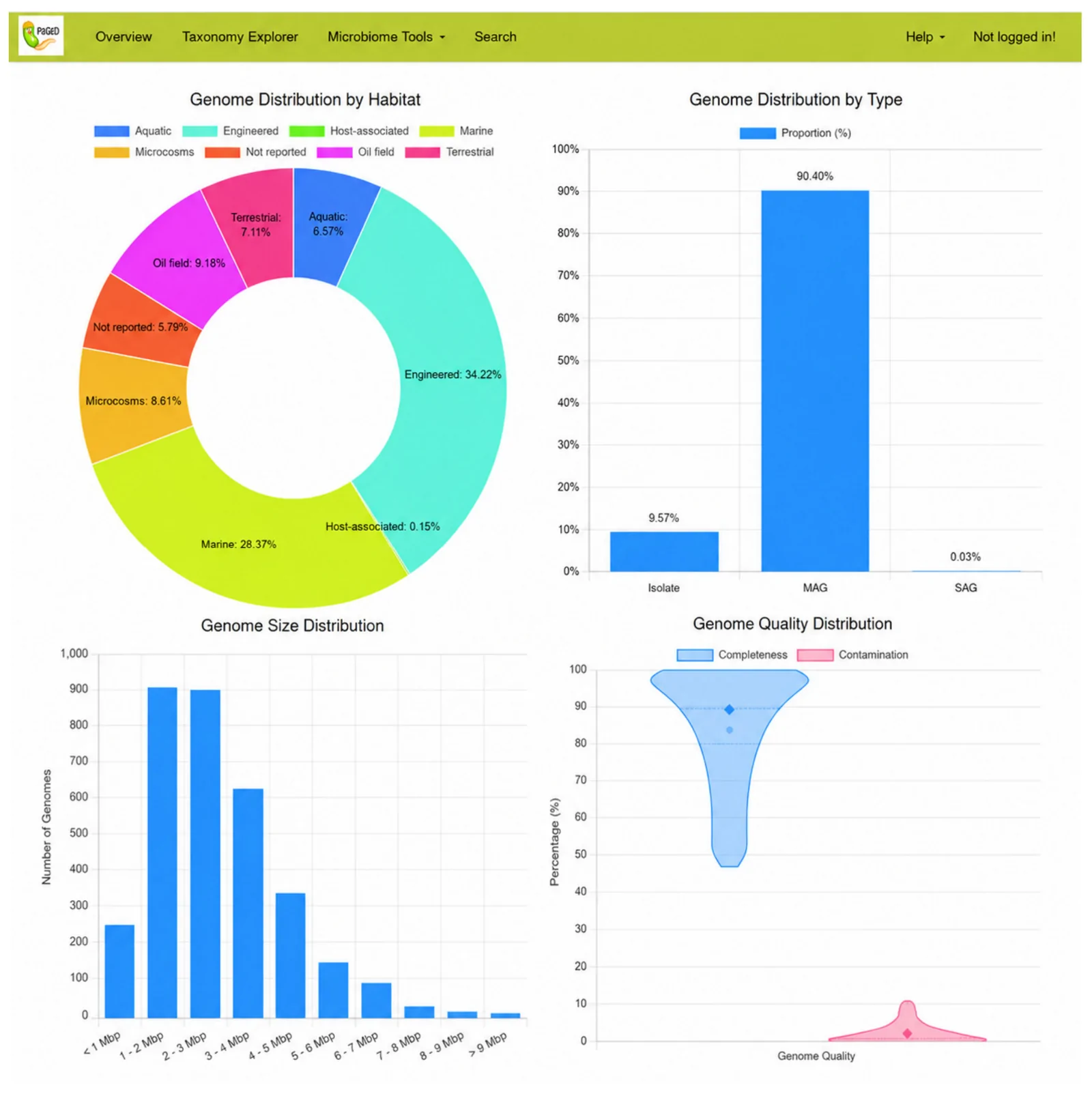
Screenshot of the PaGeD platform’s interactive dashboard showing the distribution of genomes across various habitats and the distribution of the genome types (i.e., MAGs or isolate; the visualization, generated from associated metadata, categorizes genomes by isolation source and habitat type, providing an intuitive overview of microbial diversity in oil-associated environments). The visualization includes a histogram for genome size distribution, and a violin plot summarizing quality metrics (completeness and contamination). (URL: https://paged.cpqba.unicamp.br/overview/paged_statistics/view).

Herein, the “Petroleum-Associated Genome Database” (PaGeD), a vast collection of genomes obtained from culturable microorganisms and from metagenomes (MAGs), was created and curated. The genomes of PaGeD were originally reported as related to the oil and gas industry, spanning environments such as offshore and onshore oil reservoirs, natural oil seeps, processing fluids (e.g., produced, formation and/or injection water, fracking fluids), microbiologically influenced corrosion (MIC) samples, oil spills, hydrocarbon-impacted sites or even laboratory microcosms or enrichment cultures simulating hydrocarbon contamination. These isolation sources were grouped into habitat categories (Table S2; Figure 2). Engineered systems were the most represented category including fracturing fluids, MIC samples, machinery facilities (34.22%), followed by marine-associated habitats (28.37%). This broad marine-associated category includes water- and sediment-related isolation sources, such as seawater, oil-polluted seawater, marine sediment, hydrothermal vent sediments, deep-sea water, and deep-sea sediment seepage, as detailed in Table S2.

**Figure 3:**
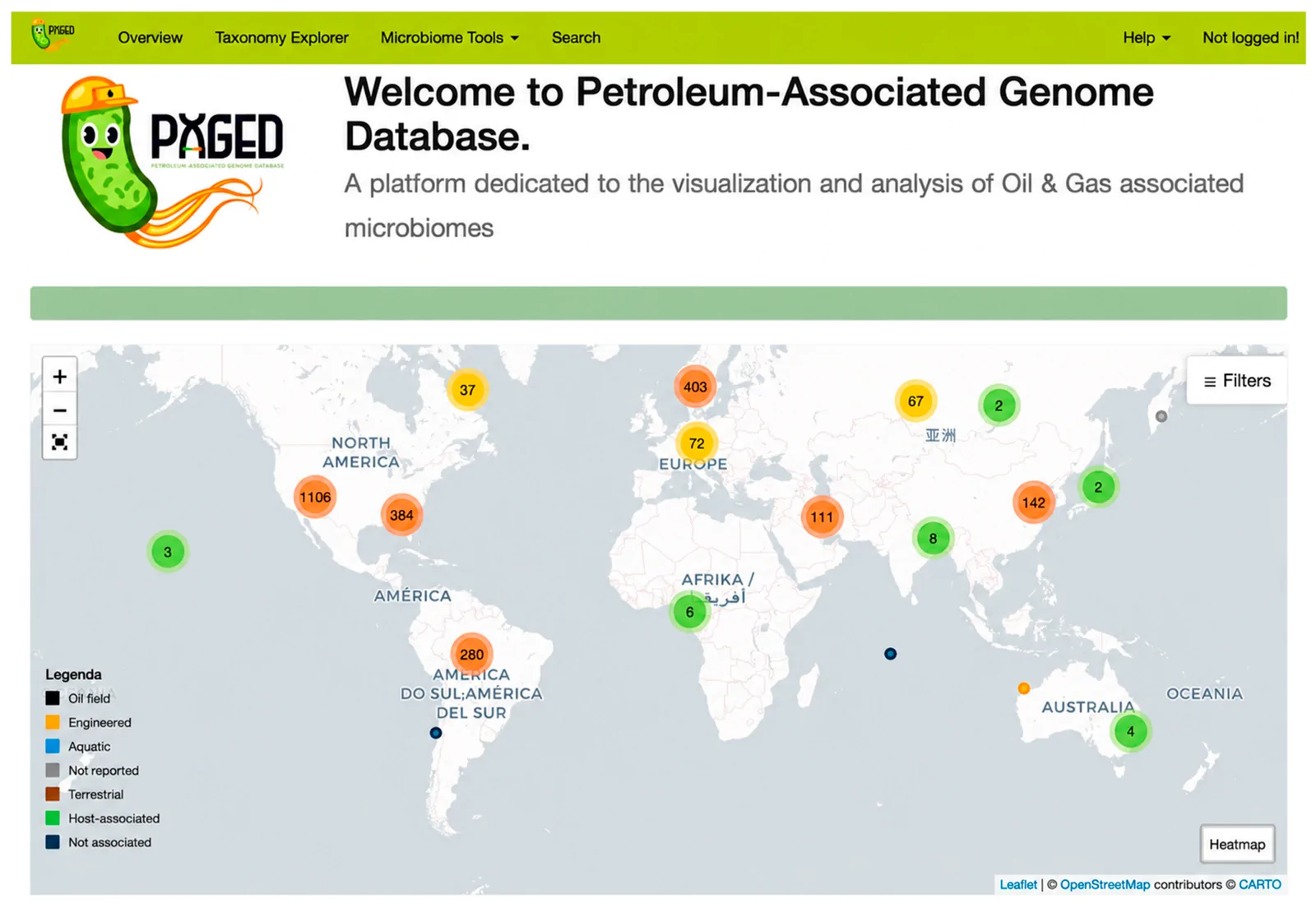
Screenshot of the PaGeD homepage displaying an interactive map of genome geographic origin across 74 countries, six continents, and major oceanic regions (URL: https://paged.cpqba.unicamp.br/).

Genomes are represented from 74 countries across six continents and major ocean basins (Figure 3). Although coverage is global, geographic representation remains uneven, with predominance of genomes from North America, particularly the Gulf of Mexico, reflecting the sequencing efforts following the Deepwater Horizon (DWH) spill [6,23,24,25,26]. As in other large-scale microbial datasets, such as the Earth Microbiome Project [27], underrepresentation of tropical regions from the Global South remains evident, highlighting opportunities for future sampling to fill geographic and ecological gaps.

### 3.2. Characterization of the PaGeD Genome Collection

The final curated dataset comprises 3334 genomes, of which 3014 correspond to MAGs and 319 are genomes derived from isolates. All genomes meet or exceed medium quality thresholds (≥50% completeness, <10% contamination). The completeness averages are 89.59 (±9.80) and 3.35% (±2.43). Approximately ~36% of the genomes contain at least partial sequences of the 16S rRNA gene.

Assembly metrics show large variation: genome sizes ranged from 0.27 Mb to 12.25 Mb, while contig counts varied from single-contig assemblies to 2899 contigs (mean = 238) (Figure 2). The smallest genomes belong to members of Nanoarchaeota and Patescibacteria (phyla known for their reduced genomes), while larger genomes were mainly affiliated with Myxococcota and Planctomycetota, phyla characterized by complex lifestyles and metabolic versatility [28,29,30].

Species-level clustering at 95% ANI led to grouping into 2522 clusters. Among these, 2089 were singletons (i.e., represented by a single genome), underscoring the high microbial diversity in petroleum-associated environments. Only 47 clusters contained five or more genomes. When compared to previous initiatives such as GROS [6], PaGeD substantially expands the known microbial diversity of oil environments. Culture-derived genomes represent only ~10% of all species-level clusters (Figure 2 and Figure S1), revealing a significant gap between cultivated and uncultivated diversity. Moreover, nearly half of the representative (centroid) genomes could not be taxonomically assigned at the genus level, highlighting the potential of PaGeD for uncovering novel microbial lineages in oil-impacted environments.

From an applied perspective, this extensive and largely unexplored diversity constitutes a valuable reservoir of metabolic potential. The species-resolved organization of PaGeD enables the systematic identification of microorganisms with relevance for oil-associated systems, supporting the screening of taxa for biotechnological applications such as bioremediation and microbial-enhanced oil recovery, as well as for microbial risk assessment. By linking genomes to habitat, geographic origin, and functional potential, PaGeD provides a practical framework that bridged large-scale genome mining with applied decision-making in environmental and industrial microbiology.

### 3.3. Phylogenetic Diversity and Potentially Novel Taxa in Oil-Associated Microbiomes

Taxonomic classification revealed the presence of 109 different phyla (94 bacterial and 15 archaeal phyla), 248 classes, 553 orders, 999 families, and 1799 genera. Pseudomonadota dominates PaGeD, with 26.7% of all genomes, followed by Bacteroidota 6.3%, Thermoproteota 5.6%, Desulfobacterota 4.8% and Chloroflexota 4.6% (Figure S1). Remarkably, PaGeD includes one class, eight orders, 36 families, 197 genera, and 437 species without any cultivated representative in the GTDB, further illustrating the contribution of PaGeD to uncovering novel lineages.

The most widely represented phylum, Pseudomonadota, was detected across all continents, diverse habitat types (e.g., marine, aquatic, terrestrial, microcosms, engineered and oil fields), and isolation sources such as hydrothermal vent sediments, microcosms, oil-polluted soils, and produced waters (Figure S2).

Notable taxa include *Acinetobacter radioresistens*, abundant in MIC-related samples, consistent with its biofilm-forming abilities in a corrosion context [31,32], and *Pseudomonas aeruginosa*, detected across a wide array of environments, reflecting its ecological versatility [33] and reinforcing its role in biosurfactant production, bioremediation and microbial-enhanced oil recovery (MEOR) [34,35]. *P. aeruginosa* exemplifies the applied relevance of specific petroleum-associated taxa.

Interestingly, nearly 3% of the genomes were affiliated with the recently proposed Patescibacteria phylum [36], with greater prevalence in activated sludge, wastewater, and tailing ponds (Figure S3). Members of this phylum are known for their ultrasmall cell size and reduced genomes, often lacking essential biosynthetic pathways, which support the hypothesis of a symbiotic lifestyle [37]. The occurrence of Patescibacteria in oil-associated environments suggests that microbial communities in such habitats may also include organisms with reduced metabolic capacities and host-dependent or symbiotic lifestyles, highlighting the complexity of microbial interactions in these systems [38].

### 3.4. Habitat Specificity of Microbial Genera in PaGeD

**Figure 4:**
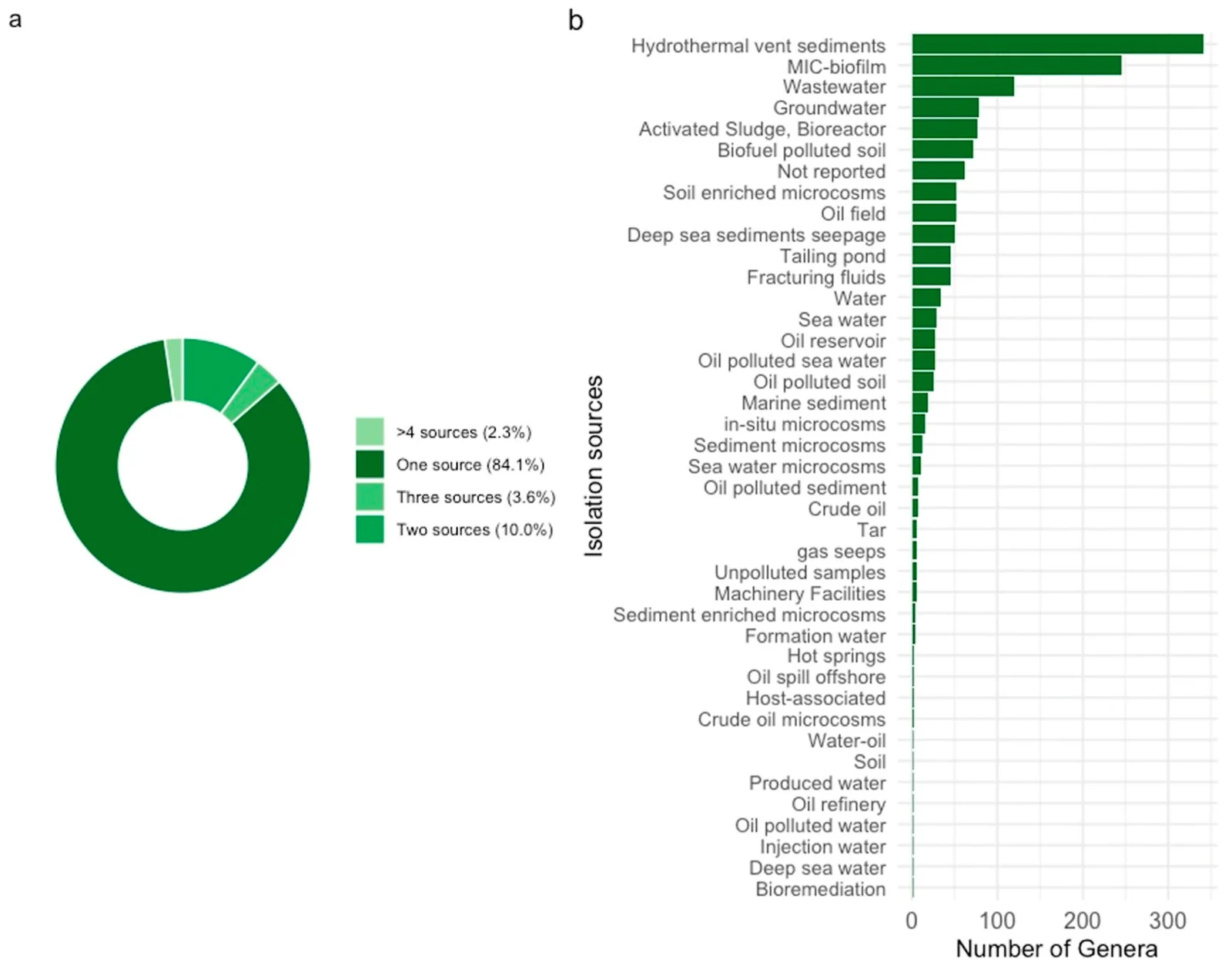
(**a**) Percentage of genera according to the number of isolation sources. (**b**) Distribution of genera found in only one isolation source according to the type of environment.

PaGeD also provides a valuable framework for exploring the ecological breadth and biogeographic distribution of oil-associated microbial genera. The distribution of genera revealed a high degree of habitat specificity within the PaGeD dataset: approximately 84% of the genera were found restricted to a single isolation source, while 10% occurred in just two sources (Figure 4a). This sharp decline in genus prevalence with increasing habitat diversity indicates that most petroleum-associated genera represented in PaGeD show source-restricted occurrence patterns, across multiple habitats, corroborating trends described in previous studies [39,40]. Importantly, this habitat-level pattern should not be interpreted as geographic endemism. Some well-known marine oil-degrading genera, such as *Alcanivorax* and *Marinobacter* [41], occur across multiple locations worldwide but remain strongly associated with marine oil-impacted environments, natural seeps or other hydrocarbon-enriched marine systems. Thus, these taxa represent exceptions to geographic restriction, while still illustrating the habitat-associated structure captured by PaGeD. Among genera restricted to a single isolation source, hydrothermal vent sediments from the Guaymas Basin emerged as the most compositionally unique (Figure 4b). This finding aligns with previous reports highlighting the distinctiveness of microbiomes in hydrothermal settings [42,43], likely driven by extreme physicochemical conditions and high levels of hydrocarbon exposure.

### 3.5. Functional Insights into Hydrocarbon Degradation Profiles of Oil-Associated Microbes

In addition to the taxonomic and ecological diversity contained in PaGeD genomes, we further investigated their functional potential with emphasis on hydrocarbon degradation. From a genomic perspective, hydrocarbon metabolism is structured as a modular process, in which chemically diverse substrates are initially activated by specialized enzymes and subsequently funneled into a limited set of central intermediates such as catechol and benzoyl-CoA, that feed into core catabolic pathways [44,45,46]. This architecture underpins both metabolic specialization and ecological cooperation, as different taxa may contribute distinct steps of the overall degradation process.

Genome-resolved approaches have revealed that enzymes responsible for the initial activation of specific hydrocarbons (e.g., alkanes, BTEX compounds, or polycyclic aromatic hydrocarbons) are often phylogenetically restricted, whereas downstream pathways involved in the processing of central intermediates are broadly distributed across bacterial and archaeal lineages [41,47]. Such functional stratification reflects evolutionary convergence toward shared metabolic solutions in hydrocarbon-impacted environments and supports the view that petroleum microbiomes are organized around a combination of specialist degraders and generalist metabolizers.

Within this framework, PaGeD provides a unique opportunity to examine how these functional modules are distributed across thousands of genomes originating from diverse petroleum-related habitats. By integrating taxonomic resolution with pathway-level annotation, the platform enables the identification of conserved catabolic cores, rare and substrate-specific pathways, and lineage-specific patterns of metabolic specialization. The analyses presented in the following sections explore this functional landscape at multiple levels, from global genomic capacities to species- and lineage-resolved hydrocarbon degradation profiles.

### 3.6. Overview of Genomic Functional Capacities

Functional annotation identified 8485 KOs across the dataset, with individual genomes containing an average of 664 ± 507 KOs. The most abundant categories were amino acid metabolism, carbohydrate metabolism, and energy metabolism (Figure S4). Stress response and quorum sensing genes were also common. Transporter families such as ABC (ATP-binding cassette) and MFS (major facilitator superfamily) were widely represented. These transport systems likely play a key role in substrate uptake and efflux mechanisms, which are crucial for microbial adaptation to hydrocarbon-rich environments [48].

**Figure 5:**
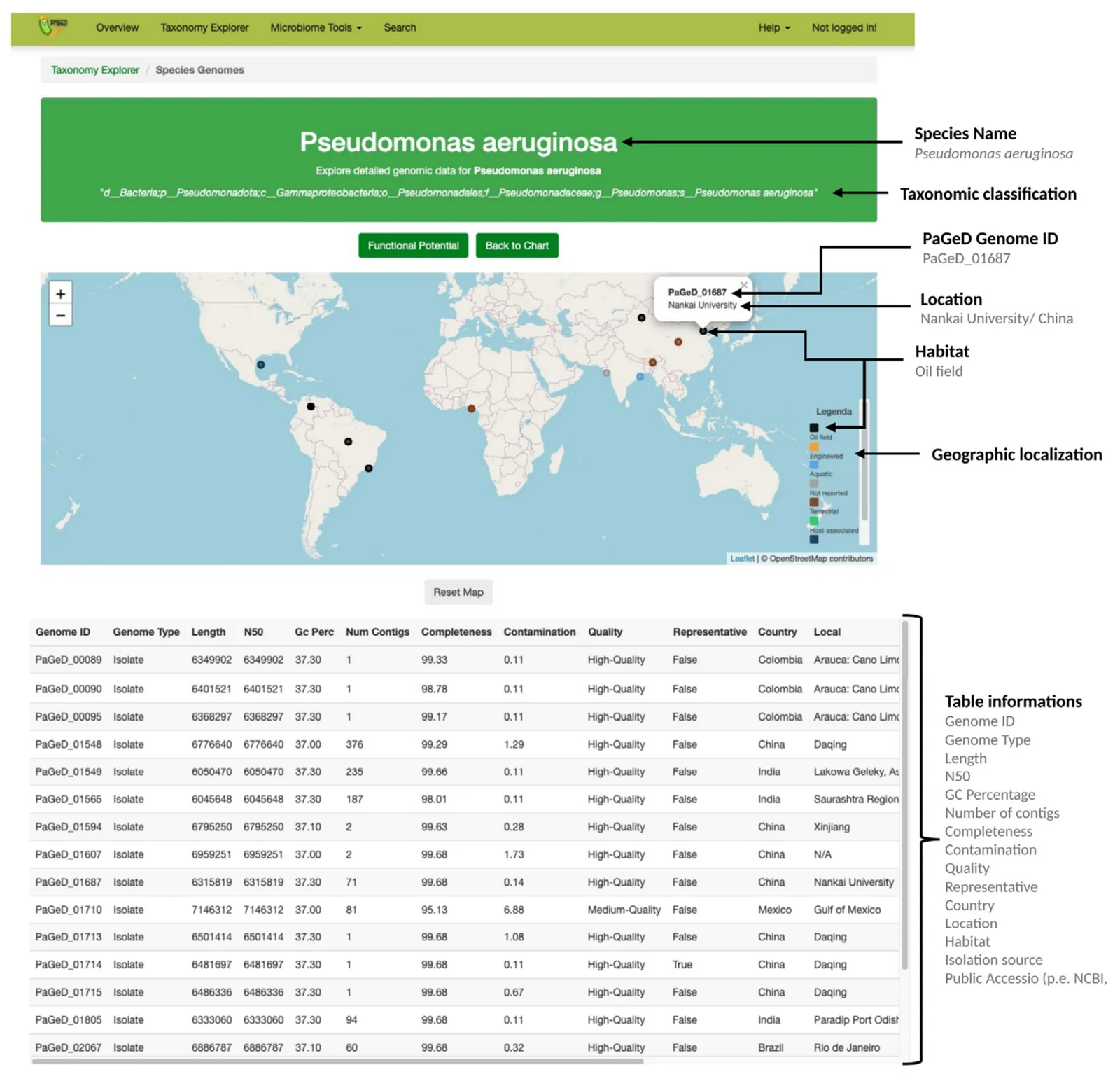
Species page for Pseudomonas aeruginosa. This page provides comprehensive information of the species, including its associated genomes, geographic distribution (displayed via an interactive map), and genome type (e.g., metagenome-assembled genomes vs. isolate genomes). Additionally, habitat details and direct links to public accession numbers (p.e NCBI, JGI) are available for further validation and exploration of the genomic data (URL: https://paged.cpqba.unicamp.br/taxonomy_explorer/species_genomes/1621).

In the PaGeD platform, users can navigate to species-specific pages that consolidate taxonomic, ecological, and functional information for each genome cluster (Figure 5). The pages integrate: (i) taxonomic classification, (ii) geographic distribution of genomes via interactive maps, and (iii) general metadata. Each genome entry also links directly to public accession numbers (e.g., NCBI, JGI), ensuring traceability and reuse. Importantly, from this page users can access additional tabs to explore the general and hydrocarbon-specific functional potential of each species.

**Figure 6:**
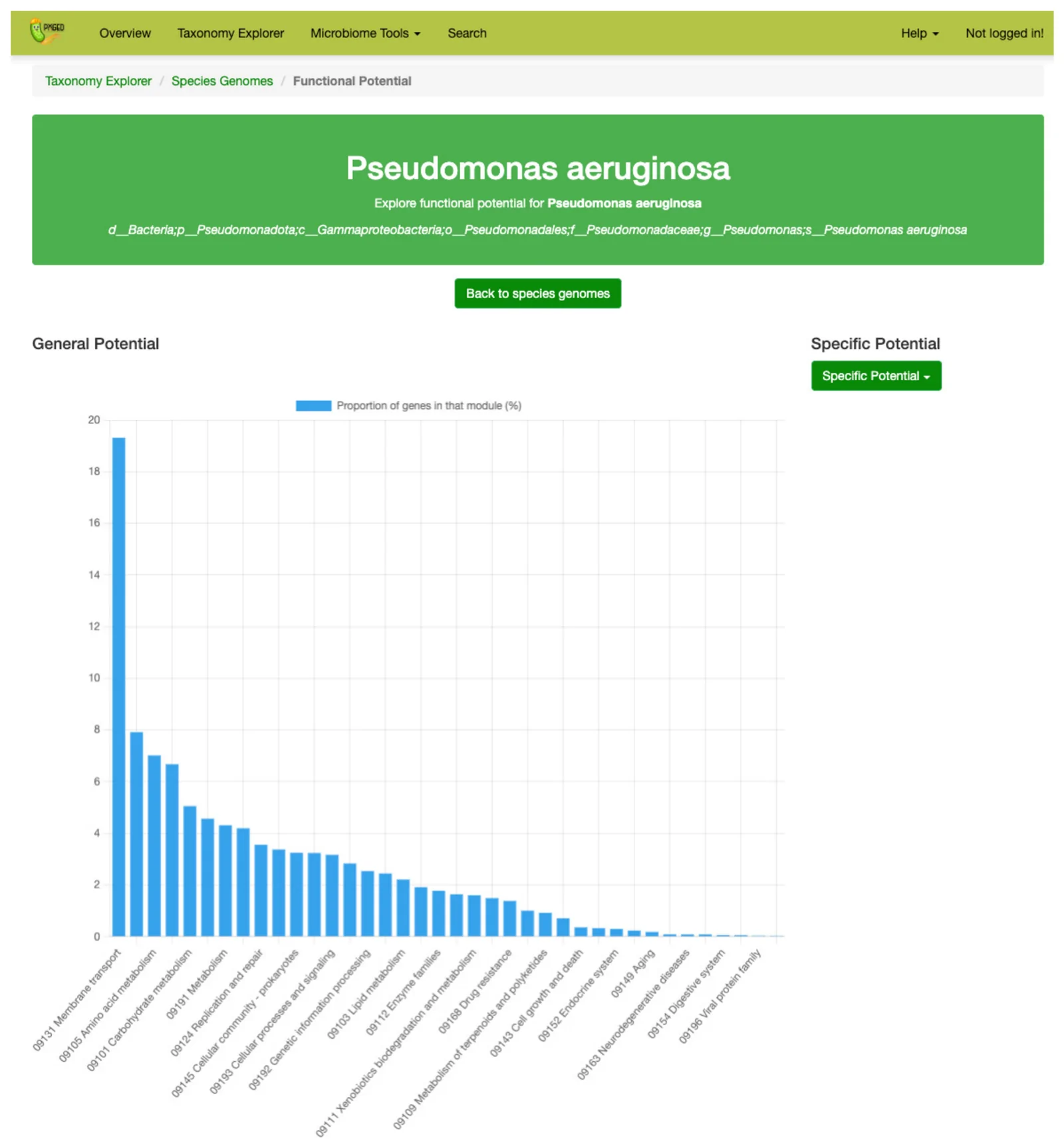
Screenshot of the PaGeD web platform displaying the General Functional Potential interface for Pseudomonas aeruginosa. The bar plot shows the relative proportion of genes assigned to KEGG Level 2 functional categories, providing an overview of the metabolic capacities of this species (URL: https://paged.cpqba.unicamp.br/taxonomy_explorer/species_genomes/1621/potential/general_potential).

While the global functional overview presented above was derived from the analyses performed for this manuscript, the PaGeD platform currently provides species-level summaries of the functional potential of the deposited genomes. For each species, the average proportion of genes assigned to KEGG Level 2 categories was calculated and is displayed as bar plots, based on all genomes available in the database. These plots offer a descriptive view of the metabolic traits of individual taxa, serving as an exploratory tool for users interested in the functional repertoire of particular species (Figure 6).

### 3.7. Functional Landscape of Hydrocarbon Degradation in PaGeD Genomes

**Figure 7:**
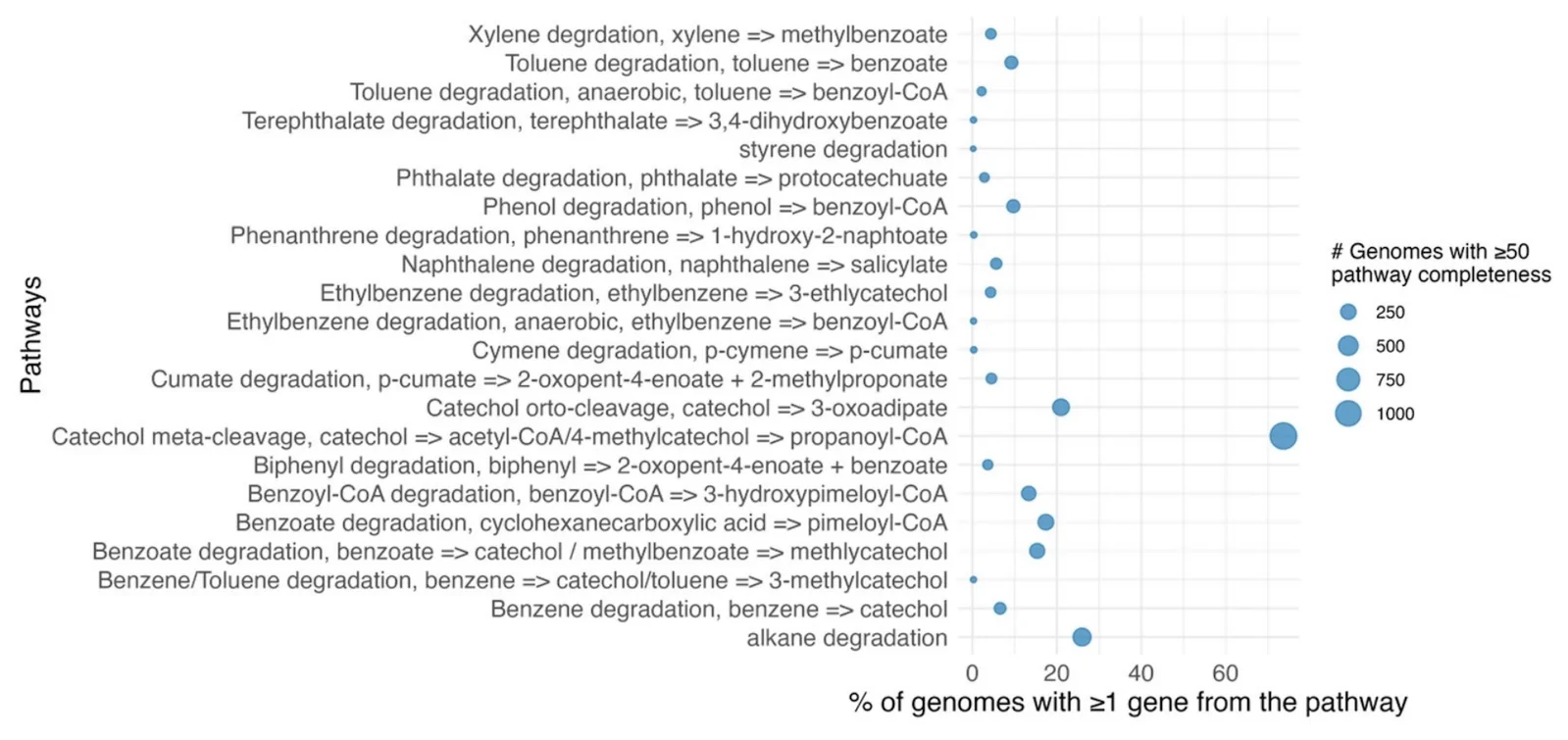
Distribution of hydrocarbon degradation pathways across genomes in PaGeD. The Y-axis lists metabolic pathways involved in hydrocarbon degradation. The X-axis shows the percentage of genomes that encode at least one gene from each pathway. Bubble size indicates the number of genomes with ≥50% pathway completeness, providing a combined view of pathway prevalence and functional richness across the dataset.

To assess the hydrocarbon degradation-related functional potential encoded within PaGeD genomes, we screened all annotated protein sequences against a manually curated list of 144 KEGG Orthology (KO) identifiers (Table S3). A total of 1505 genomes (45.1%) harbored at least one hydrocarbon degradation gene. To further characterize this functional potential, we examined the representativeness in the dataset of 22 curated metabolic modules involved in the aerobic and anaerobic hydrocarbon transformation. Central aromatic-intermediate pathways, such as catechol, benzoyl-CoA and benzoate degradation, were widespread and represented with a high completeness score (Figure 7). These highly prevalent central pathways are involved in the processing of core aromatic intermediates that may result from the initial activation of structurally diverse hydrocarbons, as well as from other aromatic compound sources. The wide distribution of these modules in PaGeD’s genomes indicates that microbial assemblages in petroleum-associated environments are enriched in conserved metabolic routes for aromatic-intermediate processing, suggesting adaptative metabolic versatility and functional connectivity within hydrocarbon degradation-related networks.

Conversely, peripheral pathways responsible for the initial activation of specific hydrocarbon classes, such as ethylbenzene, naphthalene, or phenanthrene, were identified in fewer than 5% of genomes. However, when present, they often exhibited a high completeness score, reflecting high representation of the curated KOs assigned to these modules. This pattern is consistent with a modular architecture in hydrocarbon degradation-related metabolism, in which broadly conserved central aromatic-intermediate modules are widely distributed. At the same time peripheral and substrate-specific pathways are restricted to specialized ecological niches of phylogenetic groups. Somee, M.R. et al. (2022) reported similar results; they observed a broad phylogenetic distribution of key enzymes involved in the transformation of hydrocarbon aerobic degradation intermediates, in contrast to a more limited distribution of key enzymes responsible for the initial attack of specific hydrocarbons [47].

**Figure 8:**
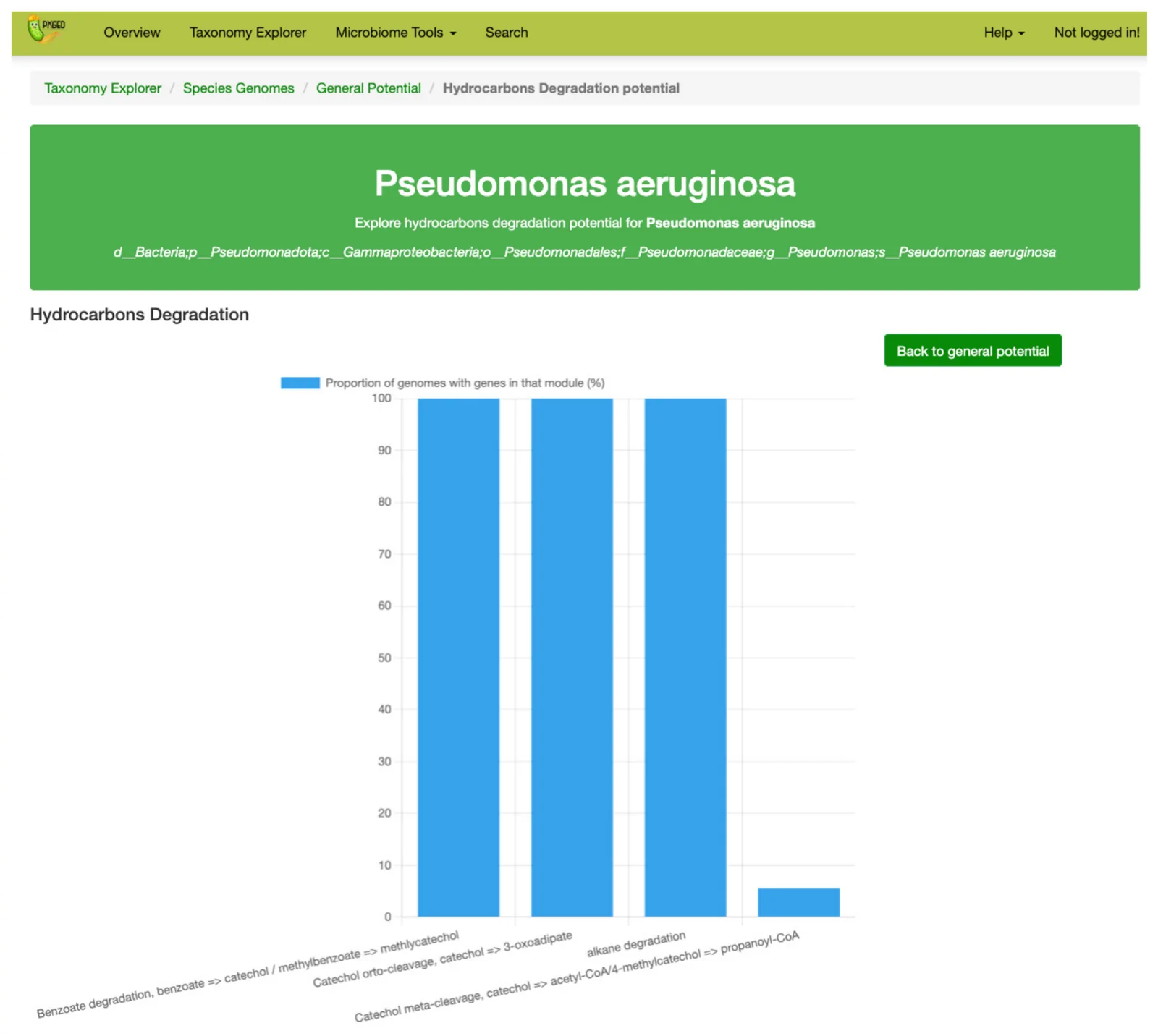
Screenshot of the PaGeD web platform displaying the Hydrocarbon Degradation Potential interface for Pseudomonas aeruginosa. The bar plot represents the completeness score of curated KEGG modules associated with hydrocarbon degradation pathways, expressed as the percentage of pathway genes encoded by this species (URL: https://paged.cpqba.unicamp.br/taxonomy_explorer/species_genomes/1621/potential/deghyd).

In addition to the general functional profiles, PaGeD also offers species-level summaries of hydrocarbon degradation-related functional potential. For each curated metabolic module, pathway/module completeness scores were calculated as the proportion of curated KOs detected for each pathway/module and then averaged across genomes belonging to the same species-level cluster. These scores represent KO-set completeness/representation and should not be interpreted as binary confirmation of fully functional pathways. The bar plots, such as the ones illustrated in Figure 8, highlight which hydrocarbon degradation-related modules are most prevalent within in each taxon, providing users with a practical tool to investigate both the broad aromatic-intermediate metabolism and substrate-specific hydrocarbon transformation potential in oil-associated microorganisms.

### 3.8. From Phyla to Genera: Taxonomic Layers of Hydrocarbon Degrading Capacity

To explore how hydrocarbon degradation-related functional potential is distributed across the microbial diversity represented in PaGeD, we calculated the average completeness of catabolic modules/pathways at both phylum (Figure S5a) and genus levels (Figure S5b). This lineage-resolved approach revealed a pronounced functional stratification, where broad central aromatic-intermediate pathways are widely conserved across multiple phyla, while peripheral and substrate-specific routes, including hydrocarbon activation and peripheral reactions, are more sporadically distributed and often restricted to specialized genera. By integrating information on pathway completeness, we uncovered clear patterns of metabolic versatility and specialization that shape the ecological roles of petroleum-associated microbes.

At the phylum level, Pseudomonadota stood out with the broadest and most complete repertoire of pathways (Figure S5a). Genomes from this phylum exhibited a high average completeness score for both peripheral pathways associated with the transformation of specific hydrocarbon classes (e.g., ethylbenzene, alkane, biphenyl) and central aromatic-intermediate modules (e.g., catechol and benzoyl-CoA degradation), highlighting their metabolic versatility and ecological dominance in oil-associated environments [41,44,47,49,50,51]. Zooming into the genus level, we observed striking heterogeneity in catabolic specialization (Figure S5b). *Pseudomonas* emerged as a central player, showing broad representativeness across nearly all modules and a particularly high average completeness score in catechol ortho-cleavage (36%) and benzoate metabolism. These findings are in accordance with several previous reports showing the ability of this genus to degrade different kinds of hydrocarbons and to metabolize a wide range of aromatic intermediates [47,52,53,54]. Several genera showed functional specialization, with high scores restricted to specific modules. For instance, *Cycloclasticus* showed a >35% completeness score in toluene and biphenyl degradation pathways, consistent with its known adaptation to PAH degradation in marine ecosystems [55,56].

The catechol meta-cleavage module, previously highlighted as the most prevalent across PaGeD, was confirmed to be broadly distributed across phyla and among a wide range of genera, including metabolically streamlined or niche-adapted groups (e.g., *Sulfitobacter*, *Enterococcus*, *Oleispira*, *Smithella*), although often with a low completeness score at the individual genome level. This finding suggests a strong ecological convergence towards the ability to process aromatic intermediates. Conversely, peripheral pathways such as naphthalene, styrene, and phthalate degradation were typically restricted to a few genera, often with a high pathway completeness score, supporting the view that specialized functions are phylogenetically restricted [47,57].

Altogether, these findings reveal a functional landscape shaped by both substrate-specific degraders and aromatic-intermediate metabolizers. Particular genera possess peripheral catabolic pathways that may enable them to initiate the degradation of specific hydrocarbon compounds (e.g., alkanes, PAHs, BTEX), whereas others appear to be more strongly associated with the downstream processing of central intermediates like catechol, benzoate and benzoyl-CoA. The widespread distribution of these central aromatic-intermediate pathways across diverse phyla and genera suggests evolutionary convergence and functional redundancy, which likely enhance ecosystem resilience and enable metabolic cooperation in petroleum-contaminated environments [47].

As an example of practical application, users interested in light-oil-associated compounds can use our search tool to query modules related to alkane and BTEX transformation, including toluene, xylene and ethylbenzene degradation, together with downstream aromatic-intermediate pathways such as catechol and benzoate metabolism. Conversely, users interested in heavier or more recalcitrant petroleum fractions may explore modules associated with PAH transformation, such as naphthalene, phenanthrene, and biphenyl degradation, as well as central aromatic-intermediate pathways including benzoyl-CoA metabolism. Thus, PaGeD provides a framework to connect petroleum-associated genome data with functional modules relevant to different hydrocarbon classes.

### 3.9. Keystone Genomes in Hydrocarbon Degradation

We identified individual genomes encoding particularly rich repertoires of degradation genes, such as *Paraburkholderia aromaticivorans*, *Cycloclasticus* spp., and *Pseudomonas veronii.* These taxa encode complete or near-complete modules for multiple aromatic pathways, highlighting their potential ecological importance (Figure S6).

**Figure 9:**
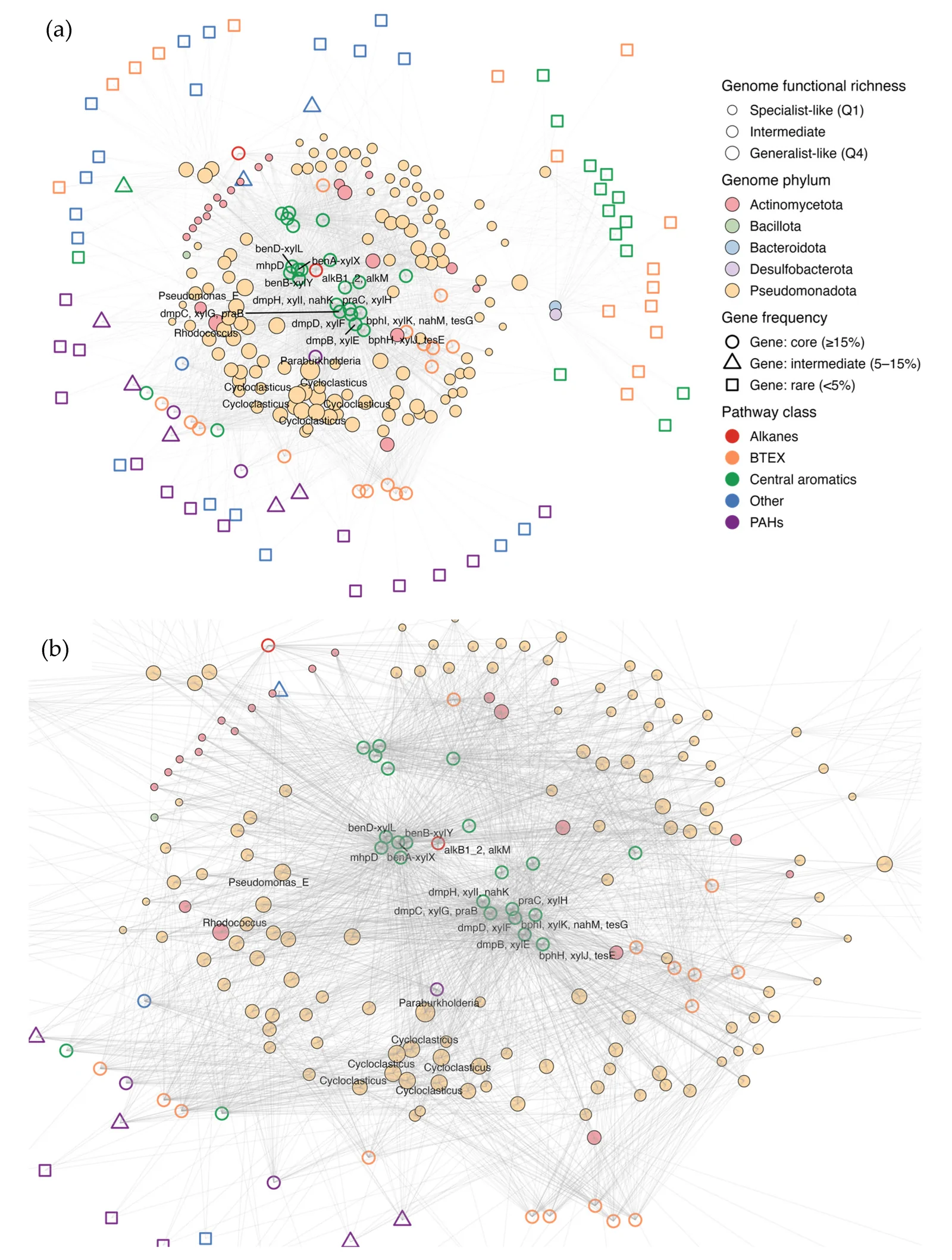
Bipartite network linking hydrocarbon degradation genes and genomes in PaGeD. (**a**) Global network showing the organization of hydrocarbon degradation functions across all genomes encoding ≥15 degradation-related KOs. Nodes represent either genomes (filled circles) or genes (hollow symbols). Genome nodes are colored by phylum and scaled by functional richness (number of hydrocarbon degradation KOs), distinguishing specialist-like (Q1) from generalist-like (Q4) genomes. Gene nodes are colored by pathway class and shaped according to their frequency across genomes (core ≥15%, intermediate 5–15%, rare <5%). Edges indicate the presence of a gene in a genome. (**b**) Zooming into the functional core of the network, highlights core genes and generalist-like genomes. This view reveals a dense hub dominated by central aromatic pathways (e.g., catechol and benzoyl-CoA degradation) and highly connected taxa such as Pseudomonas, Cycloclasticus, Paraburkholderia, and Rhodococcus. The contrast between the dense central core and the sparse periphery illustrates the coexistence of broadly conserved central catabolic functions and specialized, substrate-specific pathways. Together, these panels depict a modular architecture in which a small set of highly connected genes and genomes form a functional backbone, while specialist degraders and rare genes occupy peripheral positions.

Rather than inferring unknown gene-host associations, the bipartite network was constructed as an exploratory framework to visualize the organization of hydrocarbon functions across genomes, identify highly connected functional hubs, and examine gradients of functional specialization. The network links hydrocarbon degradation genes to the genomes encoding them (Figure 9a,b).

The resulting bipartite network comprised 143 genome nodes and 115 gene nodes connected by 2940 edges, and exhibited a low overall density (0.089), indicating selective and non-random associations between genes and genomes. The degree distribution was strongly rich-skewed (mean degree = 22.8; maximum degree = 108), revealing a small number of highly connected hubs contrasted with a large fraction of low-degree nodes. Community detection yielded a modularity of Q = 0.31, consistent with a moderately modular architecture in which specialized substructures are embedded within a shared functional backbone. Together, these properties support a network organization dominated by central catabolic genes (e.g., catechol cleavage, benzoate, and benzoyl-CoA) and generalist-like genomes, surrounded by peripheral, specialist functions. These core genes were recurrently encoded by the same genomes, forming a highly connected central backbone of the bipartite network and suggesting conserved metabolic blocks and ecological selection for shared intermediate processing [45,58].

Genomes with higher functional richness occupied more central positions in the network, whereas genomes encoding fewer degradation genes were preferentially located at the periphery. In line with the operational definitions adopted here, genomes in the upper quartile of functional richness exhibit a generalist-like profile, encoding broad repertoires of hydrocarbon degradation genes, while genomes in the lower quartile display specialist-like profiles, typically encoding only one or a few such functions.

Altogether, this topology illustrates redundancy in central modules and specialization in peripheral ones, revealing an ecological and functional complementarity that may contribute to the robustness and versatility of microbial communities in petroleum-associated environments. From an applied perspective, this structure provides a conceptual basis for identifying candidate keystone taxa and functional markers that may serve as targets for monitoring and biotechnological exploration.

### 3.10. Hydrocarbon Degradation Profiles Across Habitats

The completeness score of hydrocarbon degradation modules was further examined across habitats (Figure S7). Central modules showed consistent representation across environments, while peripheral modules displayed habitat-specific enrichment. For example, phenanthrene degradation was more prevalent in genomes from oil sand tailing ponds, consistent with selective pressures in highly contaminated engineered environments. The high prevalence of the phenanthrene module observed in PaGeD genomes from these systems may reflect the presence of such specialized degraders enriched by long-term adaptation to the recalcitrant contaminants.

A principal coordinate analysis (PCoA) based on the Jaccard distance between genomes (Figure S8) revealed limited separation among habitats, indicating a high degree of functional overlap in hydrocarbon degradation profiles across environments. Rather than reflecting the absence of ecological structure, this pattern suggests that genomes from distinct petroleum-related habitats share a substantial fraction of catabolic traits. Ordination methods based on presence–absence metrics, such as Jaccard distance, are widely used to compare functional repertoires and emphasize compositional similarity over abundance [59,60]. In this context, the lack of strong clustering implies that core hydrocarbon degradation modules are broadly distributed among genomes, regardless of their environmental origin.

This functional convergence is consistent with the idea that exposure to hydrocarbons imposes similar selective pressures across aquatic, terrestrial, engineered, and subsurface systems, favoring the retention of shared catabolic functions. At the same time, the partial dispersion observed in the ordination space reflects the presence of habitat-specific and substrate-specialized pathways, as highlighted by the differential distribution of peripheral modules (Figure S7). Together, these patterns support a model in which petroleum-associated microbiomes are structured around a common catabolic core, complemented by niche-dependent specialization, rather than forming strictly habitat-segregated functional assemblages.

### 3.11. Biogeographic Patterns of Genomes’ Hydrocarbon Degradation-Related Potential

Mapping genomes with hydrocarbon degradation-related functional potential (Figure S9) revealed geographic clusters in regions with extensive petroleum-related research and environmental impact. One prominent hotspot was observed in the Gulf of Mexico, a region heavily studied in the aftermath of the Deepwater Horizon spill, reflecting both the ecological relevance of petroleum-impacted environments and the legacy of intense genomic exploration in this area [6,23,24,61,62,63]. Another noteworthy cluster was observed in Eastern China, a region with known petroleum basins and extensive industrial activity. The presence of functionally rich genomes in this geographic region likely reflects both environmental contamination and intense research activity [64,65,66,67,68,69,70]. Despite the broad geographic coverage, underrepresentation of regions in Africa, South America, Southeast Asia and the Mediterranean persists, limiting current knowledge of petroleum-associated microbial diversity.

PaGeD should be interpreted as a curated catalog of genomic occurrences in petroleum-associated environments. Thus, each geographic datapoint indicates that a genome or genome-resolved microbial population was recovered from a given location and environmental context, rather than representing a time-resolved description of local microbiome dynamics. This distinction is particularly relevant for oil spill scenarios, where microbial communities can shift over time with changing environmental conditions.

## 4. Conclusions

This work presents the first version of the PaGeD platform and database, offering an interactive interface for further exploring genomic and associated metadata from oil-associated microbiomes. By integrating high-quality genomes with curated functional annotations, PaGeD provides an unprecedented resource to investigate the diversity, ecology, and hydrocarbon degradation potential of oil-associated microbial communities. This framework not only supports fundamental research but also has the potential to inform applied strategies in bioremediation and microbial risk management.

The database enables users to navigate species-level clusters, explore habitat and geographic distributions, and examine both general and hydrocarbon-specific functional profiles through user-friendly visualizations. These features facilitate comparative and integrative studies, offering a scalable framework that can be applied to diverse research questions in microbiology, biotechnology, and microbial risk management.

Future developments of PaGeD will include the integration of additional functional annotations of industrial and environmental relevance, such as antimicrobial and heavy metal resistance genes (resistome), mobile genetic elements, and biosynthetic gene clusters. Future updates will also aim to incorporate more detailed environmental, geochemical, and methodological metadata when available from original records or publications, including crude oil properties, salinity, heavy metals and contaminant concentrations, sequencing strategies, sample pretreatment, and other experimental details. Expanded filtering options allowing users to restrict analyses to genomes according to quality categories, including high-quality and medium-quality MAGs, will also be incorporated in upcoming versions of PaGeD, thereby enabling users to evaluate the effect of genome completeness on functional profiles. Regular updates with newly available genomes will further expand taxonomic and functional coverage, strengthening PaGeD as an open and evolving resource for the scientific community.

## Data Availability

The curated dataset underlying this study is publicly available at the Research Data Repository (REDU) under the accession DOI: https://doi.org/10.25824/redu/VKTSIB. This repository includes metadata associated with 3334 genomes compiled for the PaGeD platform, covering taxonomic classification, genome quality, assembly statistics, geographic origin, and ecological context. Table S1 provides detailed accession numbers (NCBI, JGI/IMG) and literature DOIs for genomes with publicly available references. All additional supporting information is included as Supplementary Information files accompanying this article.

## Supplementary Materials

The following supporting information can be downloaded at: https://www.mdpi.com/article/10.3390/microorganisms14102197/s1, Figure S1. Phylogenetic tree based on a concatenated alignment of 25 bacteria-archaea distributed single-copy genes from 2381 genomes of PaGeD. Figure S2. Screenshot of the PaGeD web platform showing the Taxonomy Explorer interface. Figure S3. Distribution of species-level clusters with at least five members across the isolation sources. Figure S4. Horizontal bar plot showing the number of genes annotated in each KEGG Level 2 functional category. Figure S5. Functional stratification of hydrocarbon degradation pathways across microbial taxa. Figure S6. Hydrocarbon degradation pathway completeness across the top 10 genomes with the highest catabolic gene content. Figure S7. Hydrocarbon degradation pathway completeness across the habitats. Figure S8. Principal Coordinates Analysis (PCoA) of genomes based on hydrocarbon degradation gene profiles. Figure S9. Global distribution of genomes encoding hydrocarbon degradation genes. Table S1. Genomes’ associated metadata. Table S2. Habitat and isolation source categories. Table S3. Hydrocarbon degradation gene Dataset. Table S4. Quality assembly metrics.

## Abbreviations

The following abbreviations are used in this manuscript:

PaGeD	Petroleum-Associated Genome Database
DNA	Deoxyribonucleic Acid
MAG	Metagenome-Assembled Genomes
GROS	Genome Repository of Oil Systems
IMG/M	Integrated Microbial Genomes & Microbiomes
NCBI	National Center for Biotechnology Information
JGI/IMG	Joint Genome Institute/Integrated Microbial Genomes & Microbiomes
tRNA	Transfer Ribonucleic Acid
GTDB-tk	Genome Taxonomy Data Base Tool kit
ANI	Average Nucleotide Identity
ORF	Open Reading Frame
KO	KEGG Orthology
MIC	Microbiology-Influenced Corrosion
DWH	Deepwater Horizon
rRNA	Ribosomal Ribonucleic Acid
MEOR	Microbial-Enhanced Oil Recovery
BTEX	Benzene, Toluene, Ethylbenzene and Xylenes
ABC	ATP-Binding Cassette
MFS	Major Facilitator Superfamily
PCoA	Principal Coordinate Analysis
