# Supplementary material for: Conserved Hydrocarbon Degradation Architectures in Petroleum-Associated Microbiomes Revealed by Genome-Resolved Analysis Using the Petroleum-Associated Genome Database (PaGeD)
Source: Microorganisms. 2026 Oct 1;14(10):2197. doi: 10.3390/microorganisms14102197 (PMC13707590; doi:10.3390/microorganisms14102197)
Supplement: Supplementary file 1 [file microorganisms-14-02197-s001.zip › Hidalgo_et_al_2026_supplementary_material_microorganisms_version.pdf]

microorganisms

MDPI

Supplementary Materials

Conserved hydrocarbon degradation architectures in petroleum-associated microbiomes revealed by genome-resolved analysis using the Petroleum-Associated Genome Database (PaGeD)

Kelly Hidalgo-Martinez [1,*], Renato Augusto Corrêa dos Santos [2, 3, 4], João Vitor Leite Novoletti [5], Tulio de Lucca Capelini [1], Ana Letícia Malheiros Oliveira [6], Diego Mauricio Riaño-Pachón [2, 3, 4], German Zafra [7], Maira Paula de Sousa [8], Luiz Fernando Martins [8], Valéria Maia de Oliveira [1,*]

1 Divisão de Recursos Microbianos, Centro Pluridisciplinar de Pesquisas Químicas, Biológicas e Agrícolas (CPQBA), Universidade Estadual de Campinas – UNICAMP, Av. Alexandre Cazellato, 999, CEP 13148-218, Paulínia, SP, Brasil
2 Laboratory of Computational, Evolutionary, and Systems Biology, Center for Nuclear Energy in Agriculture, University of São Paulo, Piracicaba, São Paulo, Brazil; renatoacsantos@gmail.com
3 Research Centre for Greenhouse Gas Innovation – RCG2I, São Paulo, SP, Brazil; diego.riano@cena.usp.br
4 National Institute for Science and Technology of Bioethanol, São Paulo, SP, Brazil;
5 Laboratory of Isotopic Ecology. Center for Nuclear Energy in Agriculture, University of São Paulo, Piracicaba, São Paulo, Brazil; joao.novoletti@gmail.com
6 Programa de pós-graduação em Genética e Biologia Molecular, Instituto de Biologia, Universidade Estadual de Campinas (UNICAMP), Rua Monteiro Lobato 255, Cidade Universitária CEP 13083-862, Campinas, SP, Brasil
7 Grupo de Investigación en Bioquímica y Microbiología (GIBIM), Escuela de Microbiología, Universidad Industrial de Santander, Cra 27 calle 9, 680002, Bucaramanga, Colombia; geralzaf@uis.edu.co
8 PETROBRAS/ R&D Center (CENPES), Av. Horácio Macedo, 950. ZIP 21941-915 Cidade Universitária, Ilha do Fundão, Rio de Janeiro, Brazil; mpsousa@petrobras.com.br; martinslf@petrobras.com.br
* Correspondence: KJH-M: kellyjhm@unicamp.br ; VMO: vmaia@unicamp.br
Tel.:+5519981721510 (KJH-M))

microorganisms

Supplementary material content

Figure S1 3

Figure S2 4

Figure S3 5

Figure S4 6

Figure S5 7

Figure S6 8

Figure S7 9

Figure S8 10

Figure S9 11

microorganisms

MDPI

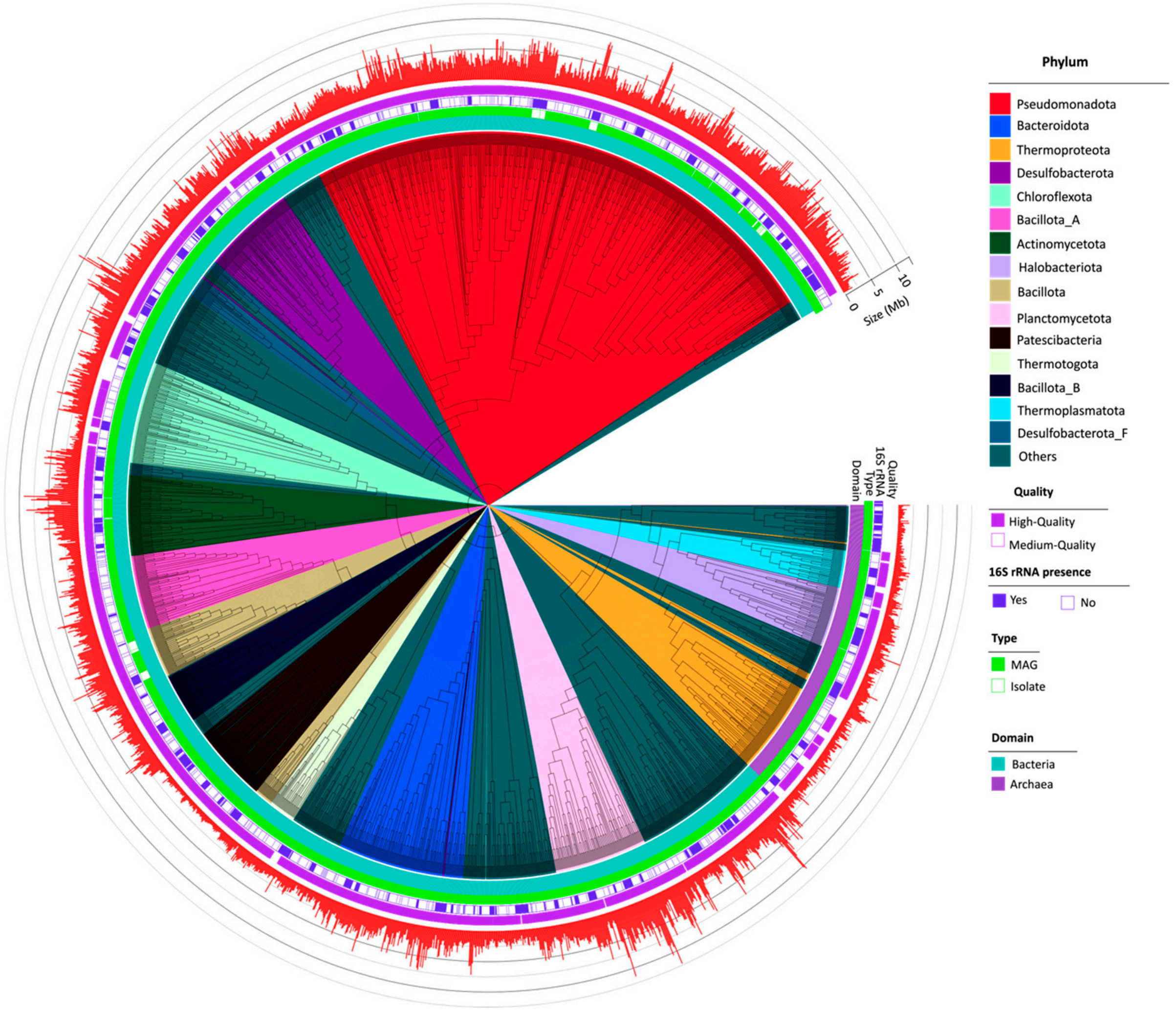

Figure S1. Phylogenetic tree based on a concatenated alignment of 25 bacteria-archaea distributed single-copy genes from 2,381 (at least 30% of the markers genes present) genomes of PaGeD.

microorganisms

MDPI

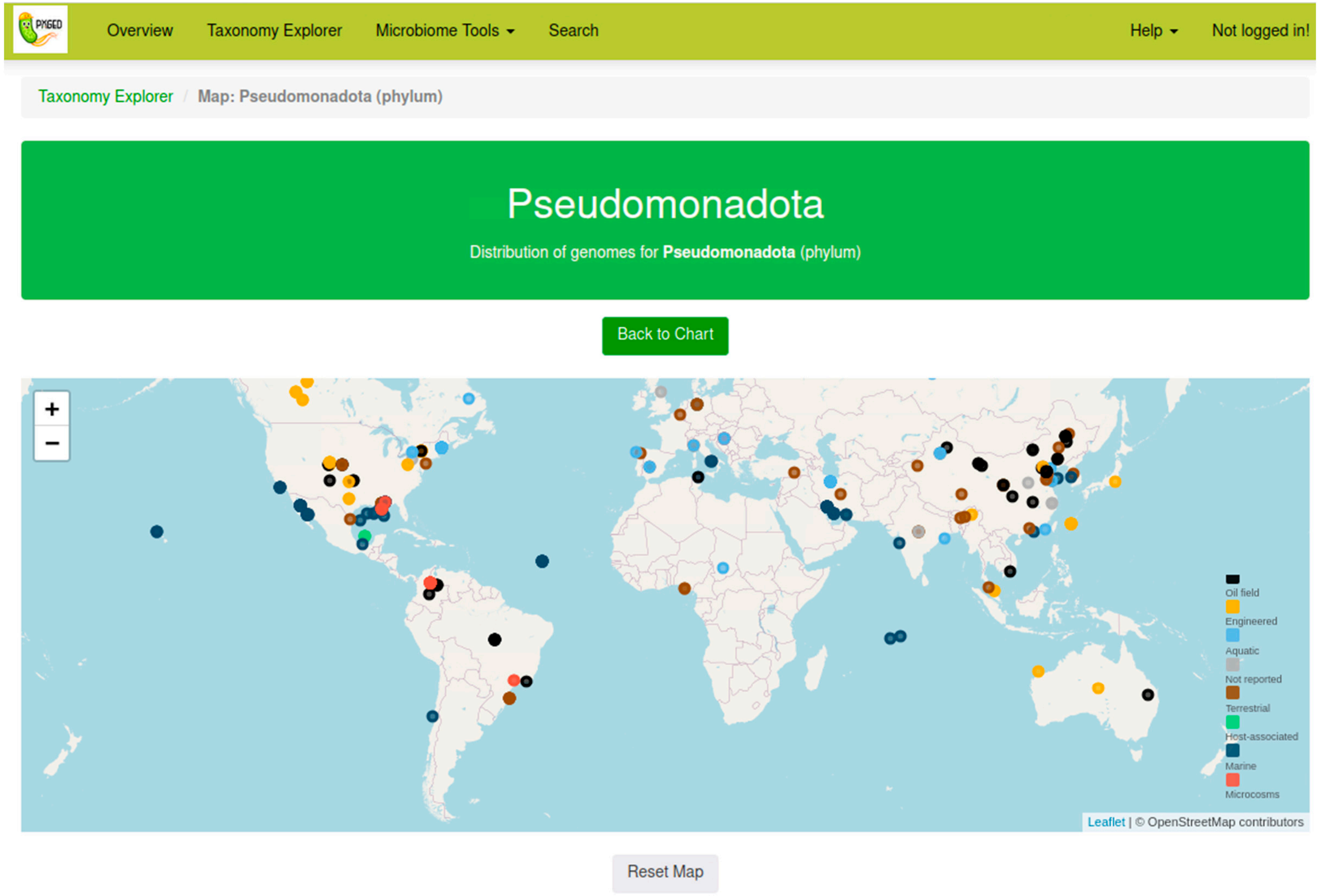

Figure S2. Screenshot of the PaGeD web platform showing the Taxonomy Explorer interface. The map displays the global geographic distribution of genomes classified under the phylum Pseudomonadota. Genomes are color-coded by habitat category, and interactive features allow users to zoom, pan, and explore detailed metadata for each genome (URL: https://paged.cpqba.unicamp.br/taxonomy_explorer/taxon_map/phylum/Pseudomonadota).

microorganisms

MDPI

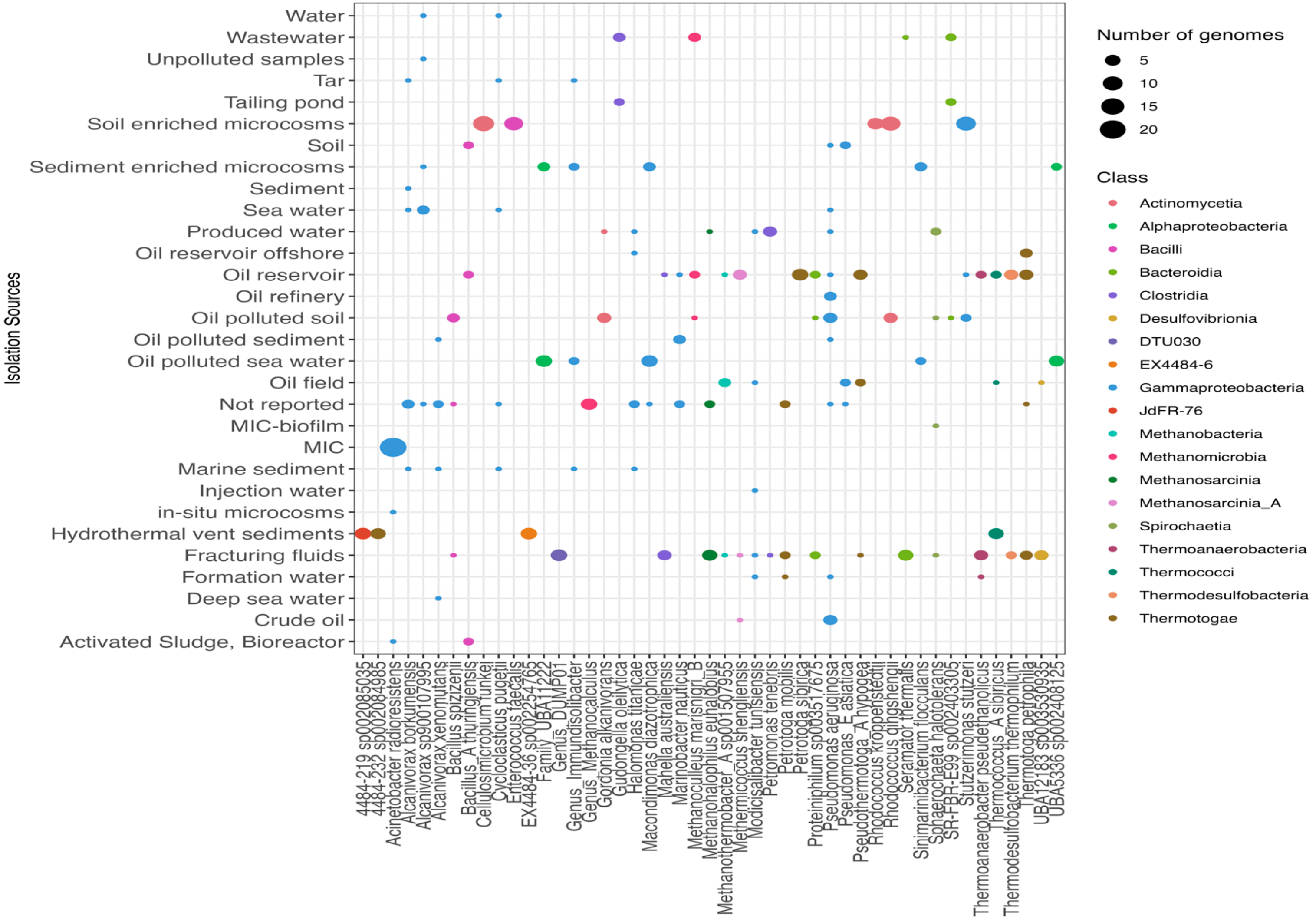

Figure S3. Distribution of species-level clusters with at least five members across the isolation sources. The bubble colors represent the class taxonomic level, and the size represents the number of genomes in each cluster. Only species-like clusters with at least five genomes were plotted.

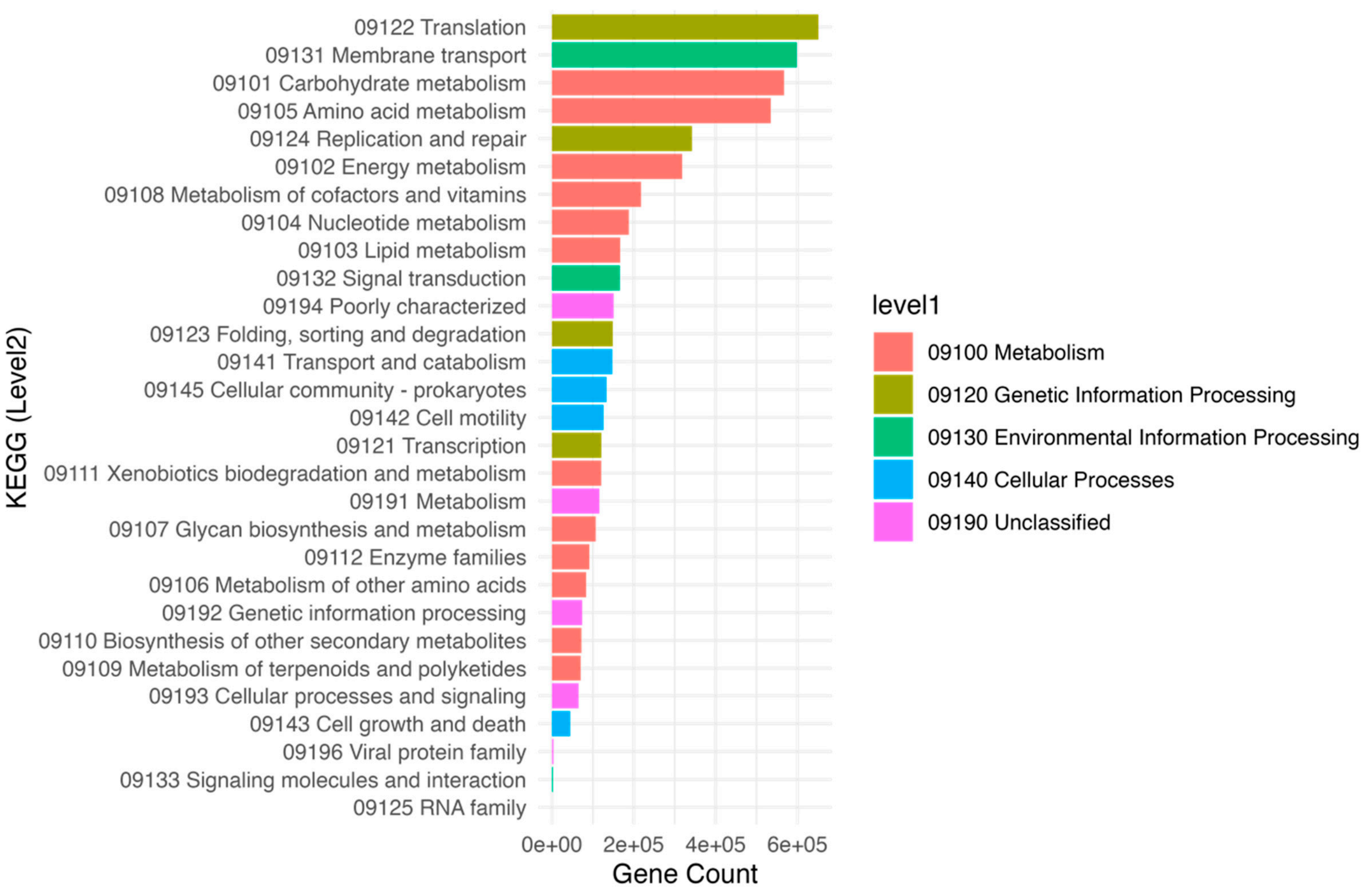

Figure S4. Horizontal bar plot showing the number of genes annotated in each KEGG Level 2 functional category (Y-axis). Bars are colored according to their corresponding KEGG Level 1 category, as indicated in the legend.

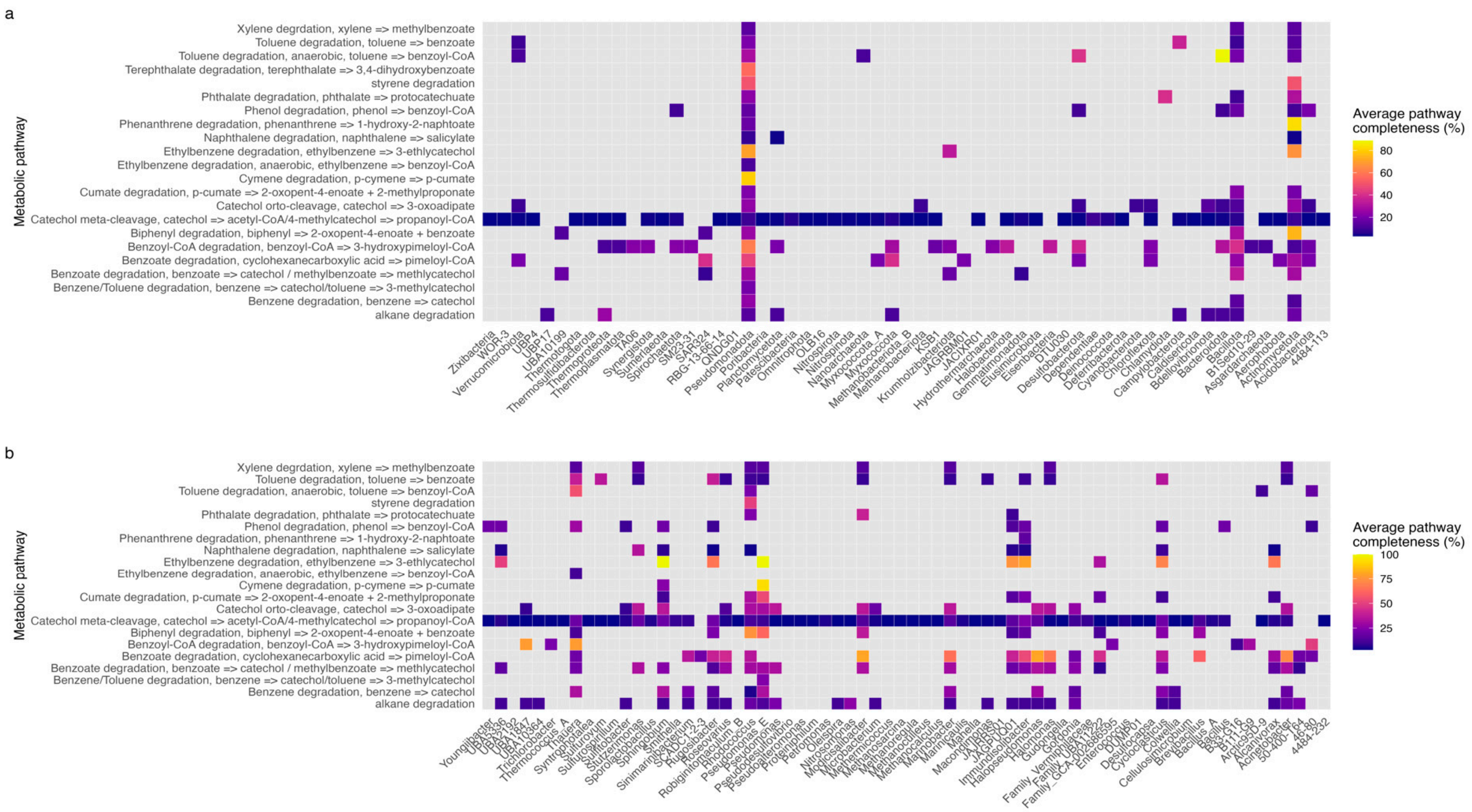

Figure S5. Functional stratification of hydrocarbon degradation pathways across microbial taxa. Average completeness of each metabolic pathway across (a) phyla and (b) genera.

microorganisms MDPI

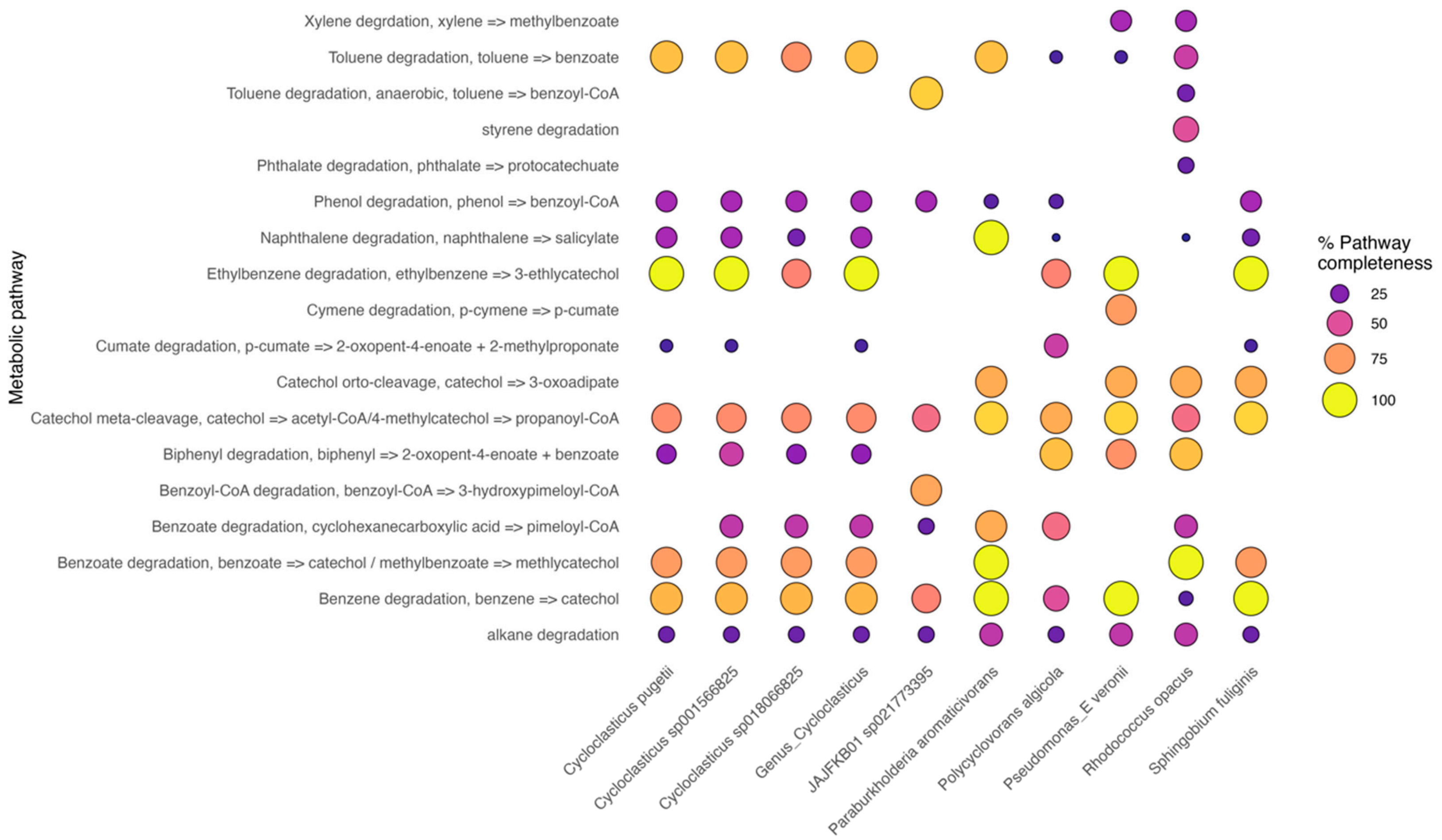

Figure S6. Hydrocarbon degradation pathway completeness across the top 10 genomes with the highest catabolic gene content. Each row represents a degradation pathway, and each column corresponds to one of the top-ranking genomes. The size and color intensity of the bubbles indicate the completeness percentage of each pathway within each genome.

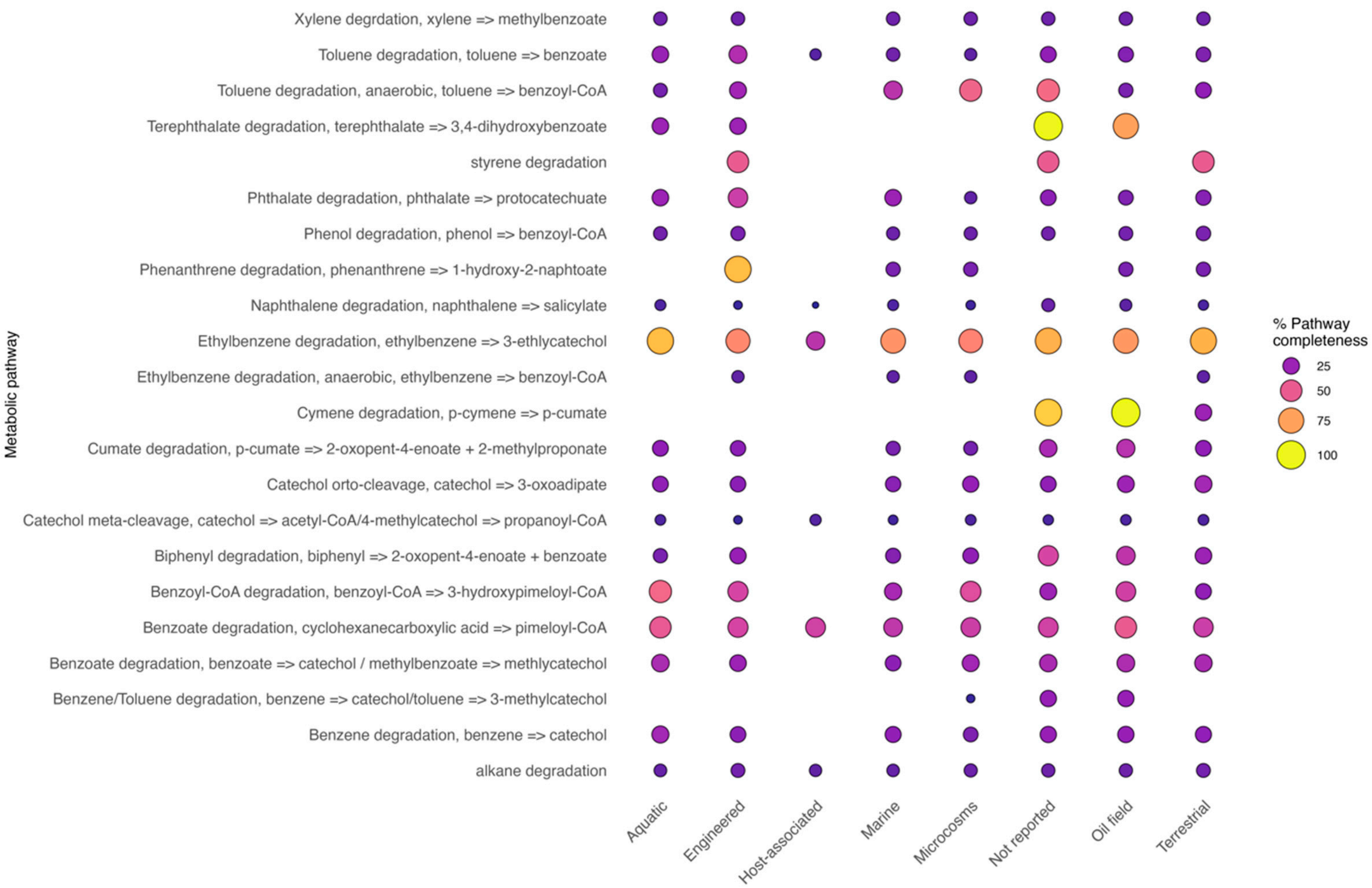

Figure S7. Hydrocarbon degradation pathway completeness across the habitats. Each row represents a degradation pathway, and each column corresponds to one habitat. The size and color intensity of the bubbles indicate the average completeness percentage of each pathway within each habitat.

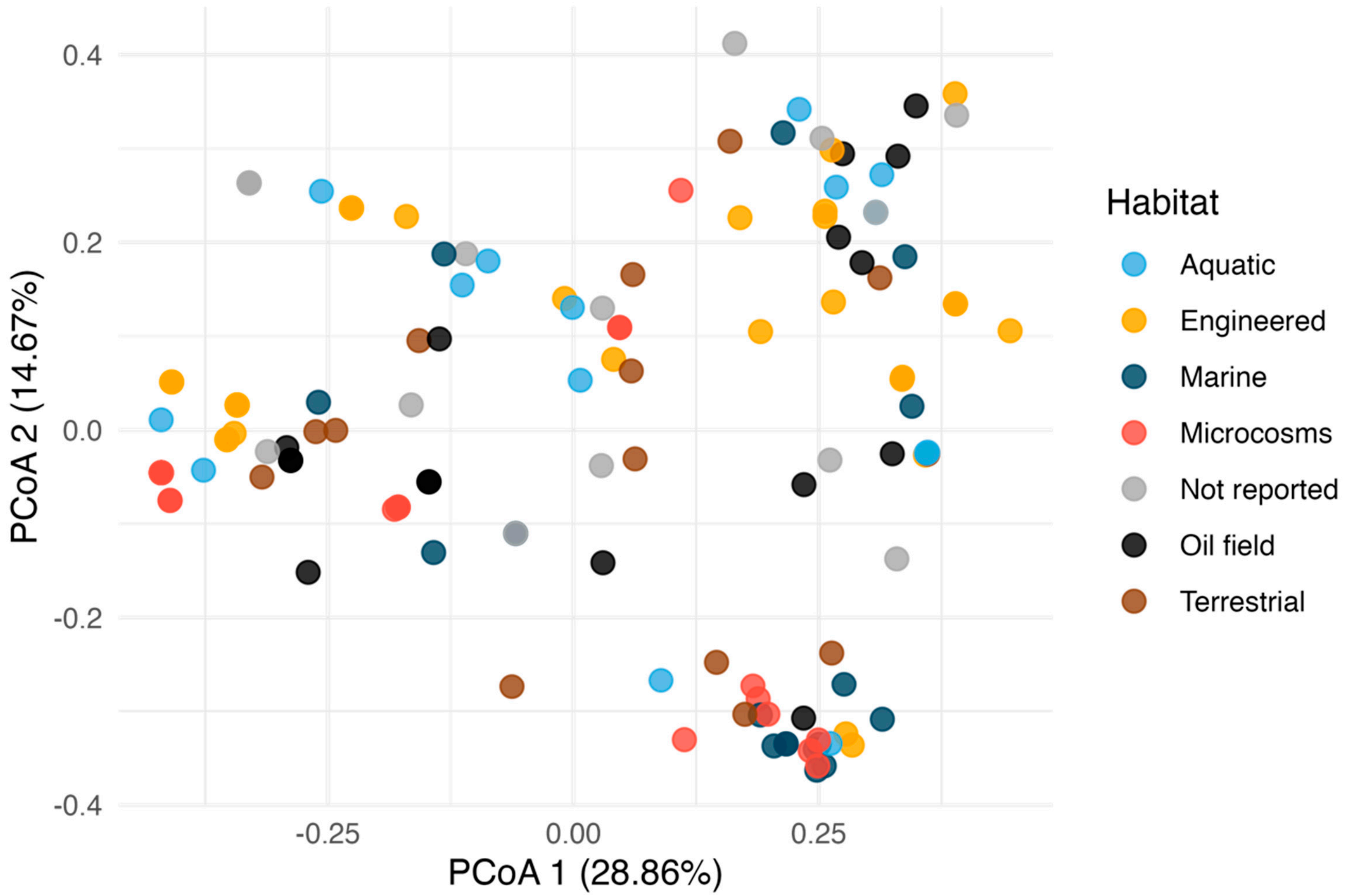

Figure S8. Principal Coordinates Analysis (PCoA) of genomes based on hydrocarbon degradation gene profiles. The ordination is based on Jaccard distance using the presence-absence matrix of curated degradation genes. Each point represents a genome containing at least 15 hydrocarbon degradation genes, colored by its associated habitat.

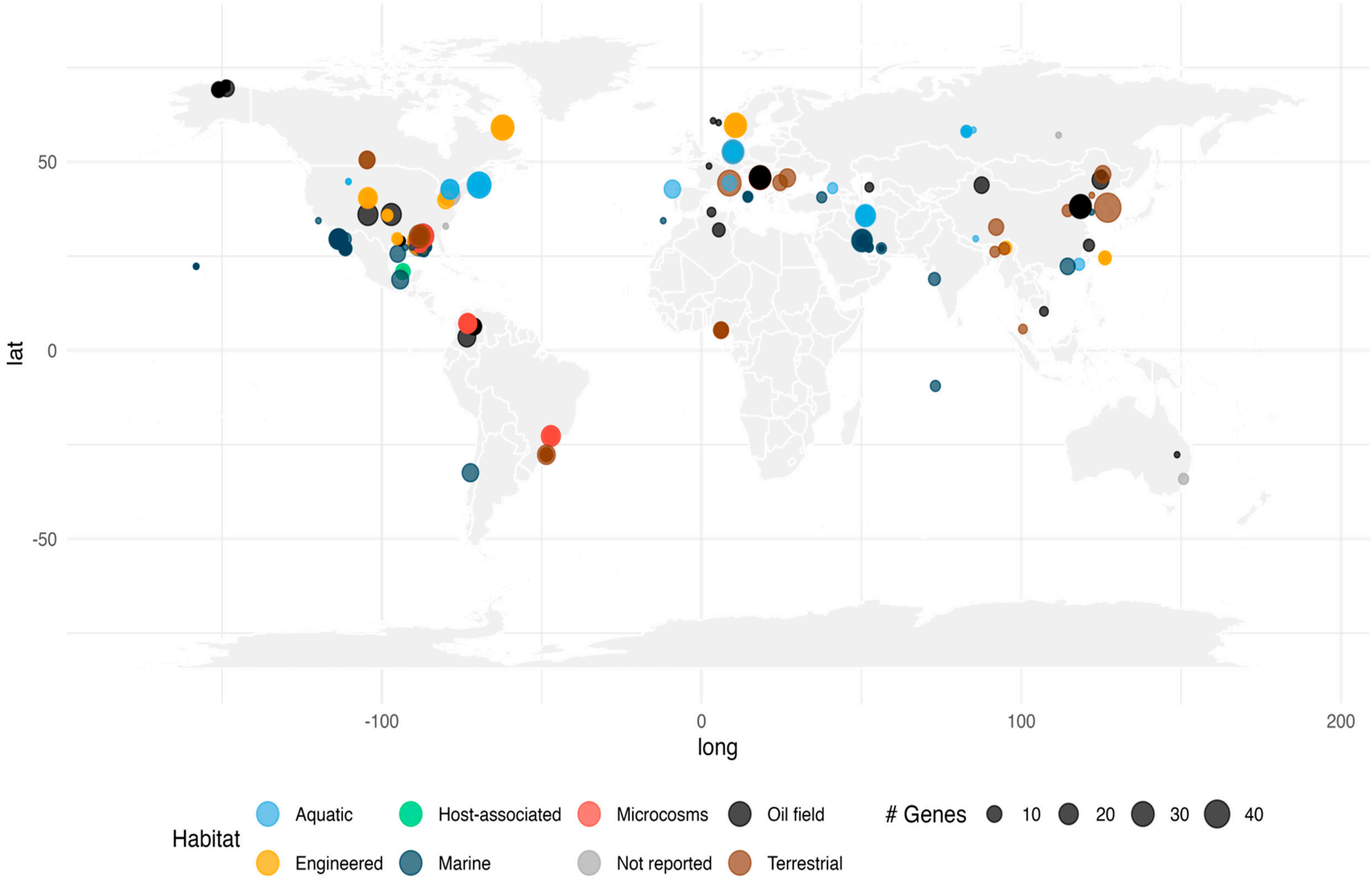

Figure S9. Global distribution of genomes encoding hydrocarbon degradation genes. Each point represents a genome mapped to its geographic origin. Point size reflects the number of hydrocarbon degradation genes annotated in the genome, while colors indicate the associated habitat category.
